# Reducing alcohol use through alcohol control policies in the general population and population subgroups: a systematic review and meta-analysis

**DOI:** 10.1016/j.eclinm.2023.101996

**Published:** 2023-05-10

**Authors:** Carolin Kilian, Julia M. Lemp, Laura Llamosas-Falcón, Tessa Carr, Yu Ye, William C. Kerr, Nina Mulia, Klajdi Puka, Aurélie M. Lasserre, Sophie Bright, Jürgen Rehm, Charlotte Probst

**Affiliations:** a Institute for Mental Health Policy Research, Centre for Addiction and Mental Health, Toronto, ON, Canada; b Heidelberg Institute of Global Health (HIGH), Medical Faculty and University Hospital, Heidelberg University, Heidelberg, Germany; c Alcohol Research Group, Public Health Institute, Emeryville, CA, United States; d Department of Epidemiology and Biostatistics, Western University, London, ON, Canada; e Addiction Medicine, Department of Psychiatry, Lausanne University Hospital, Lausanne, Switzerland; f School of Health and Related Research (ScHARR), Faculty of Medicine, Dentistry & Health, University of Sheffield, Sheffield, England, UK; g Center for Interdisciplinary Addiction Research (ZIS), Department of Psychiatry and Psychotherapy, University Medical Center Hamburg-Eppendorf (UKE), Hamburg, Germany; h Department of Psychiatry, University of Toronto, Toronto, ON, Canada; i Campbell Family Mental Health Research Institute, Centre for Addiction and Mental Health, Toronto, ON, Canada; j Dalla Lana School of Public Health & Department of Psychiatry, University of Toronto, Toronto, ON, Canada; k Program on Substance Abuse & WHO Collaborating Centre, Public Health Agency of Catalonia, Barcelona, Spain; l I.M. Sechenov First Moscow State Medical University (Sechenov University), Moscow, Russian Federation

**Keywords:** Alcohol policy, Alcohol consumption, Effectiveness, Socioeconomic status, Race, Ethnicity

## Abstract

We estimate the effects of alcohol taxation, minimum unit pricing (MUP), and restricted temporal availability on overall alcohol consumption and review their differential impact across sociodemographic groups. Web of Science, Medline, PsycInfo, Embase, and EconLit were searched on 08/12/2022 and 09/26/2022 for studies on newly introduced or changed alcohol policies published between 2000 and 2022 (Prospero registration: CRD42022339791). We combined data using random-effects meta-analyses. Risk of bias was assessed using the Newcastle–Ottawa Scale. Of 1887 reports, 36 were eligible. Doubling alcohol taxes or introducing MUP (Int$ 0.90/10 g of pure alcohol) reduced consumption by 10% (for taxation: 95% prediction intervals [PI]: −18.5%, −1.2%; for MUP: 95% PI: −28.2%, 5.8%), restricting alcohol sales by one day a week reduced consumption by 3.6% (95% PI: −7.2%, −0.1%). Substantial between-study heterogeneity contributes to high levels of uncertainty and must be considered in interpretation. Pricing policies resulted in greater consumption changes among low-income alcohol users, while results were inconclusive for other socioeconomic indicators, gender, and racial and ethnic groups. Research is needed on the differential impact of alcohol policies, particularly for groups bearing a disproportionate alcohol-attributable health burden.

**Funding:**

Research reported in this publication was supported by the 10.13039/100000027National Institute on Alcohol Abuse and Alcoholism of the 10.13039/100000002National Institutes of Health under Award Number R01AA028009.

**Research in context**

**Evidence before this study**

Despite the effectiveness of key alcohol control policies being widely agreed on, meta-analyses on their impact on alcohol use relying on real-world evidence are surprisingly scarce. Through systematic searches on Web of Science, Medline, PsycInfo, Embase, and EconLit, we identified existing reviews published between 2000 and 2022 (*n* = 36; alcohol taxation: 14, minimum unit pricing (MUP): 2, temporal availability: 10, multiple policies: 10) that indicated clearly that increases in alcohol excise taxes, the introduction of MUP, and restrictions of temporal alcohol availability were mostly associated with a reduction in alcohol consumption. However, few of these reviews have quantified this association, and none have systematically studied the differential impact of alcohol control policies by sociodemographic factors such as gender, socioeconomic status, and race and ethnicity.

**Added value of this study**

The current study reviews the evidence put forth by a total of 36 studies published since January 2000. Through rigorous steps in extracting comparable data, we provide a systematic quantification of those studies and estimated the impact of three major alcohol control policies on alcohol consumption levels. In addition, all available evidence on alcohol policy effects conditional on gender, socioeconomic status, and race and ethnicity were summarised narratively. The greatest reduction in alcohol consumption was seen following the introduction of pricing policies, particularly for the most affordable alcohol. Based on a limited number of available studies (*n* = 9), we found that following the introduction of pricing policies, alcohol consumption reduced the most among low-income groups, while the evidence was inconclusive for other sociodemographic factors.

**Implications of all the available evidence**

Taxation increases, the introduction of MUP, and restrictions of temporal availability all contribute to a decline in consumption levels and consequently alcohol-attributable harm. However, more research is needed on their impact on health inequalities between different sociodemographic groups. Alcohol control policies need to be systematically evaluated with respect to their potential contribution to mitigate health inequalities through differential effects on alcohol use.

## Introduction

Alcohol use is among the leading risk factors for premature mortality, with an increasing number of alcohol-attributable premature deaths over the last two decades globally.^1^ This trend is partly driven by an accelerated increase in alcohol-attributable deaths among disadvantaged populations in many countries, such as people of low socioeconomic status (SES). In the United States (US), for example, alcohol-related harms contribute to a widening gap in life expectancy between men and women of low and high SES.^2^ Given alcohol's role in growing health inequalities, tackling alcohol use goes beyond reducing alcohol's harm and contributes to achieving various United Nations Sustainable Development Goals (SDG),^3^ including a reduction of inequalities (SDG target 10.2) by addressing health disparities.^4^ To reverse trends of increasing alcohol-attributable harms and health inequalities, alcohol control policies shown to reduce alcohol use and related harms in a cost-effective manner play a key role.

The World Health Organisation (WHO) recommends several alcohol policy interventions to effectively and cost-effectively lower alcohol consumption levels and related harms in society.^5^ Three policies, the so-called “best buys”, stand out in not only being the most cost-effective policies, but also relatively easy to implement: increasing alcohol prices through taxation or minimum pricing, restricting the temporal and spatial availability of alcoholic beverages, and bans and restrictions of alcohol marketing.^6^, ^7^, ^8^ However, two key questions have not yet been addressed in prior reviews: First, a quantification of their impact on consumption in real-world settings is pending, although their effectiveness, particularly in reducing alcohol-attributable harm, has been well established (for the most recent systematic reviews, see^9^, ^10^, ^11^). Second, while systematic differences in both the drinking patterns and the related harms have been observed across both SES^12^^,^^13^ and racial and ethnic groups,^14^^,^^15^ there has been minimal investigation into whether these policies can mitigate against growing inequalities in the alcohol-attributable disease burden through differential effects on alcohol use.^16^

Drawing on experiences from countries that have implemented alcohol control policies within the past decades, we conducted a systematic review and meta-analysis to quantify the effects of alcohol control interventions on alcohol consumption. Specifically, we focused on those policies implemented at the state or national level, which seem to yield almost immediate results:^17^

(1) alcohol taxation (levying an excise tax on alcoholic beverages),
(2) minimum pricing or minimum unit pricing (MP/MUP; setting a floor price for alcoholic beverages in general or for a certain amount of pure alcohol, respectively), and
(3) temporal availability of alcoholic beverages (restricting the hours per day or days per week to purchase alcoholic beverages).

As a secondary objective, we evaluated whether the intervention effects are conditional on sociodemographic factors (i.e., gender, SES, and race and ethnicity). We also considered, where reported, specific effects for any intersectional groups defined by combinations of these factors (e.g., women of low SES).

## Methods

This systematic review is reported in accordance to the Preferred Reporting Items for Systematic Reviews and Meta-Analyses (PRISMA) guideline.^18^ The review protocol was registered at Prospero (CRD42022339791) and this study addresses the first research question specified in the protocol. For departures from the protocol, see appendix p. 2.

### Search strategy and selection criteria

The following databases were searched: Web of Science; Medline, PsycInfo, and Embase via Ovid; and EconLit via EBSCO. Systematic literature searches were conducted on August 12th and September 26th, 2022 to identify eligible research reports using predefined search terms (see appendix p. 3, 5). The search was limited to original research reports published since January 1st, 2000 and no language restrictions were applied.

After removal of duplicates by the review platform Covidence,^19^ titles/abstracts (step one) and full-texts (step two) were screened by two independent reviewers (CK, LLF, and/or TC). Eligible original research reports were interventional or observational studies investigating the effectiveness of the aforementioned alcohol control policies on either individual-level or population-level alcohol consumption, including changes in the prevalence of alcohol use, and drinking patterns, within the general population or subgroups of interest (i.e., gender, SES, and/or race and ethnicity). Reports were further required to compare the intervention with a baseline or reference scenario. Reports were excluded if they were not informed by quantitative data (e.g., opinion pieces), examined short-term interventions related to specific events (e.g., sporting events or Christmas), or included multiple interventions without mutual control. There was moderate to substantial agreement between reviewers (Cohen's kappa for title/abstract screening: 0.44–0.60, full-text screening: 0.58–0.60).^20^

The systematic search was complemented by manually screening the reference lists from prior reviews on this topic (see appendix p. 7) and a grey literature search (see appendix p. 9).

The following data was extracted by CK: study characteristics (i.e., study type, data sources, number of observations, sampling design, if applicable), study location, alcohol policy, year of policy implementation, alcohol use assessment, years of follow-up, comparator (if applicable, e.g., country or territory where no policy was implemented), effect size (i.e., change in alcohol consumption), and measures of uncertainty. The extracted data of a random subset of *n* = 16 reports were independently checked by JML, revealing no inconsistencies.

### Risk of bias assessment

Risk of bias (ROB) was evaluated using the Newcastle–Ottawa Scale for assessing the quality of nonrandomised studies in meta-analysis,^21^ which was customised for the purpose of this study. The assessment was completed by two independent reviewers (CK, CP, JML, and/or KP). Cases of disagreement were resolved via team discussion. We differentiated between low, moderate, and critical ROB, with the overall assessment reflecting the most critical among all categories of assessment (i.e., selection, comparability, and outcome; for details, see appendix p. 10).

### Statistical analysis

#### Determining relative changes in consumption

The primary outcome of interest was the relative change in the level of alcohol consumption (percentage change) following each policy intervention. For studies reporting absolute consumption changes, relative change was estimated by dividing the absolute change in consumption following the intervention by the average pre-intervention consumption, over a maximum period of five years. For studies reflecting current alcohol users only, the relative change in consumption in the general population was determined under the assumption that the prevalence of alcohol use remained the same pre and post intervention. As a secondary outcome, we summarised changes in consumption patterns (e.g., changes in the alcohol use or heavy episodic drinking prevalence) narratively (results available in the appendix p. 18).

#### Comparability of policy interventions

The aggregation of the effect sizes for the different policy interventions required comparability across studies. We therefore applied the following transformations: For alcohol taxation, tax changes were converted to reflect the percentage change in the excise tax. For studies on tax elasticities, elasticity estimates were rescaled to reflect a 10% tax increase. For MP/MUP, we calculated the price per 10 g of pure alcohol in international dollars. Finally, to combine studies investigating a restriction of temporal alcohol availability with those evaluating liberalisation, we assumed that their effects on alcohol consumption are bi-directional. In other words, we assumed that liberalising availability (i.e., allowing Sunday sales) would lead to an equal absolute but *inverse* change in consumption, as would a restriction of the same (i.e., a Sunday sales ban). In a sensitivity analysis, we evaluated this assumption by including only those studies that investigated the same direction of the policy effect (see appendix p. 19, 24).

#### Quantitative summary

Meta-analyses were conducted if data from at least three independent studies were available for (a) changes in overall alcohol consumption (objective 1) or (b) changes conditional on sociodemographic factors (objective 2). For alcohol taxation, we computed a random-effects meta-regression model to test for a linear association between changes in taxation (independent variable) and consumption (dependent variable). To account for the clustered nature of the data, as some studies tested multiple associations, a study identifier was added as a random intercept and the restricted maximum-likelihood method for estimation was used.^22^ Moreover, the z-standardised location- and time-specific gross domestic product purchasing power parity (GDP PPP, per capita) in international dollars^23^ was added as a covariate, to account for income as a key determinant of affordability.^24^ In sensitivity analyses, we repeated the meta-regression model using (a) the study identifier as a covariate and the DerSimonian-Laird estimator,^22^ (b) studies on tax increases, and (c) studies with a longitudinal study design only.

To evaluate the impact of MP/MUP and temporal availability restrictions on alcohol consumption, we used random-effects meta-analyses using inverse variance weighting. Standard errors served as source of variance and were estimated if not reported (for details, see appendix p. 11). In sensitivity analyses, we used similar models or multi-level random-effects meta-regression models without intercept to explore the potential impact of covariates, such as the assessment of alcohol use (categorial: individual-level vs. aggregated data), study design (categorial: longitudinal vs. (repeated) cross-sectional) and study quality (categorial: critical vs. non-critical risk of bias).

Importantly, in the quantitative summary, we focused on the direct effect of a (beverage-specific) policy on the (beverage-specific) change in alcohol consumption. We further distinguished between studies that tested the immediate policy effect on consumption, defined as any consumption changes within one year of policy implementation, and those that looked at longer-term consumption changes, defined as any consumption changes after the first year of policy implementation.

Between-study heterogeneity was evaluated using Cochran's Q and the *I*^2^ statistic, with *I*^2^ > 50% considered substantial heterogeneity. If substantial between-study heterogeneity was observed, we tested whether the exclusion of any individual study would modify the point estimate significantly using leave-one-out analyses. Additionally, prediction intervals were calculated reflecting the uncertainty of estimates.^25^ Potential publication bias was examined based on visual inspections of funnel plots or Egger's regression-based test at a significance threshold of *p* = 0.05 (for meta-analysis only). All analyses were conducted in R version 4.2.1,^26^ using the ‘metafor’ package (version 3.8-1).^27^

#### Qualitative summary

If data from at least three independent studies were not available, results relevant to our objectives were summarised narratively. Moreover, cross-beverage policy effects, i.e., the effects of a beverage-specific policy on the consumption of beverages that were not targeted by the policy (e.g., the impact of a beer tax increase on wine consumption), were also presented in the qualitative summary, if reported.

### Role of the funding source

Research reported in this publication was supported by the 10.13039/100000027National Institute on Alcohol Abuse and Alcoholism of the 10.13039/100000002National Institutes of Health under Award Number R01AA028009. The content is solely the responsibility of the authors and does not necessarily represent the official views of the National Institutes of Health.

## Results

**Fig. 1:**
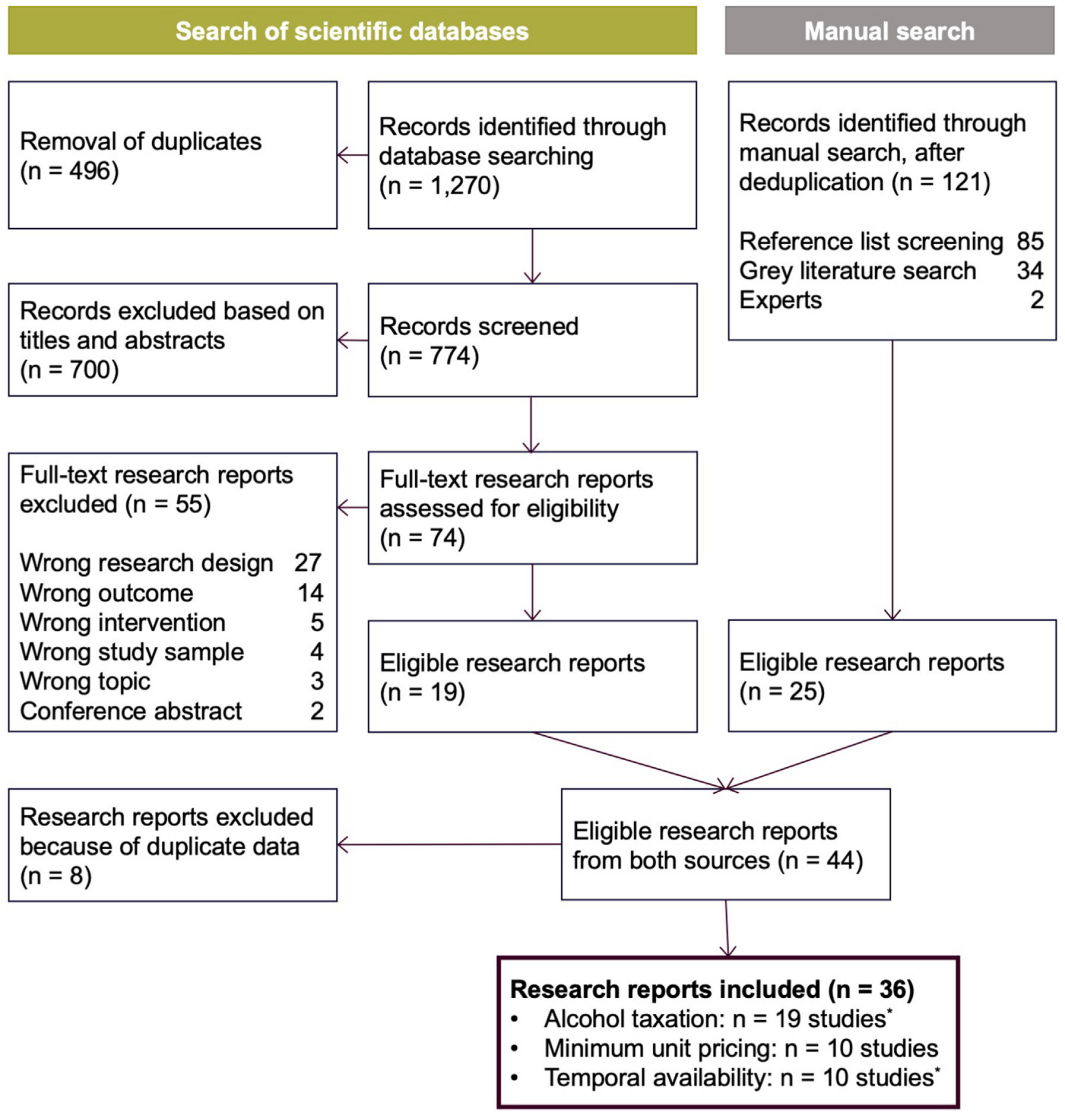
Flowchart of study selection. Note: List of excluded full-text articles is available in the appendix p. 12. ^∗^Numbers do not add up, as three studies reported results on both the impact of alcohol taxation and temporal availability policies.

Of the 1887 research reports identified in the literature search, 36 reports were eligible for inclusion (see Fig. 1). These reports covered more than 25 policy interventions in 14 countries (see appendix p. 15). Reports used either longitudinal (individual-level cohort or panel data: *n* = 8; aggregated data representing a full assessment of alcohol use at the population level: *n* = 18), repeated cross-sectional (*n* = 8), cross-sectional (*n* = 1), or mixed study designs (longitudinal and repeated cross-sectional; *n* = 1). Table 1 provides an overview of each report's study characteristics. In the following, we present the key findings of the quantitative and qualitative summary for each policy for the general population and subgroups of interest (for results on consumption patterns, see appendix p. 18).

**Table 1:** Overview and study characteristics of reports included in the systematic review.

Reference	Country (region)	Study period	Study design and data sources	Intervention	Outcome	Subgroup analysis	Risk of bias (selection/comparability/outcome)^a^
**Alcohol taxation**							
Alexeev et al. 2021^28^^,^^b^	Australia	2002–2018	Longitudinal cohort study; comparative interrupted time series analysis of the Household Income and Labour Dynamics in Australia survey (n = 223,977; age: 15+; women: not reported)	70% increase in alcohol tax on spirits-based RTD beverages in 2009	Immediate and long-term effect on alcohol use (standard drinks per day)	.	Critical (1/1/1)
Chikritzhs et al. 2009^29^	Australia	2008	Longitudinal, aggregated data; descriptive presentation of alcohol consumption data over time	70% increase in alcohol tax on spirits-based ready-to-drink beverages in 2009	Immediate effect on monthly per capita consumption	.	n. a.
Doran et al. 2011^30^	Australia	2004–2009	Longitudinal, aggregated data; descriptive presentation of alcohol sales data over time	70% increase in alcohol tax on spirits-based RTD beverages in 2009	Immediate effect on annual per capita consumption)	.	n. a.
Vandenburg et al. 2019^31^^,^^b^^,^^c^	Australia	1989–2016	Longitudinal, aggregated data; interrupted time series analysis of beer consumption data from the Australian Tax Office	Tax reform with varying nominal taxes for beer based on alcohol content and container type in 2000–2002	Immediate and long-term effect on monthly domestic beer sales	.	Critical (2/0/3)
Chung et al. 2013^32^	China (Hong Kong)	2006–2011	Repeated cross-sectional data analysis (individual-level data; n = 15,698; age: 18–70; women: 50.0–54.5%)	Elimination of excise tax on beer and wine in 2008	Immediate effect on lifetime and past-year prevalence of alcohol use, prevalence of binge drinking	.	Critical (1/0/1)
Rehm et al., 2022a^17^	Estonia, Latvia, Lithuania, Poland	2001–2020	Longitudinal, aggregated data; time-series analysis using total per capita consumption data from the World Health Organization	Various tax increases between 2001 and 2020	Immediate effect on per capita consumption	.	Moderate (2/1/3)
Tran et al. 2022^33^^,^^b^	Lithuania	2001–2019	Longitudinal, aggregated data; panel data analysis of per capita consumption data from Statistics Lithuania	Beverage-specific tax increases in 2007 and 2017	Immediate effect on annual per capita consumption	.	Critical (1/0/3)
Khaltourina et al. 2015^34^	Russia	1998–2013	Longitudinal, aggregated data; descriptive presentation of alcohol production and sales data over time	Spirits tax not adjusted to hyperinflation in 1998–1999	Immediate effect on annual retail sales of vodka and liquor	.	n. a.
Heeb et al. 2003^35^^,^^b^	Switzerland	1999–1999	Longitudinal cohort study of current alcohol users (n = 1347; age: 15+; women: 48.6%)	Tax reform to unify alcohol tax on domestic and foreign spirits in 1999	Immediate effect on beverage-specific alcohol use (grams of pure alcohol per day)	Gender	Critical (0/0/1)
Kuo et al. 2003^36^	Switzerland	1999–2001	Longitudinal cohort study of current alcohol users (n = 2923; age: 15+; women: 55.3%)	Tax reform to unify alcohol tax on domestic and foreign spirits in 1999	Immediate effect on spirits consumption (grams of pure alcohol per day)	Gender, education, employment status	Critical (1/0/1)
Sornpaisarn et al. 2013^37^^,^^b^	Thailand	2004–2009	Longitudinal, aggregated data; interrupted time-series analysis of alcohol production data from the Excise Department of Thailand	Three consecutive beverage-specific tax increases in 2005, 2007 and 2009	Immediate effect on monthly per capita alcohol production	.	Critical (1/0/3)
An et al. 2011^38^^,^^b^	USA	1984–2009	Repeated cross-sectional data analysis of the Behavioural Risk Factor Surveillance System (n = 3,932,943; mean age: 44.9 (SD: 17.7); women: 60.7%)	.	Level of alcohol use (standard drinks per month among current alcohol users), past-month alcohol use prevalence	Race/ethnicity	Critical (1/2/1)
Dávalos et al. 2012^39^	USA	2001–2005	Longitudinal cohort study of the National Epidemiological Survey on Alcohol and Related Conditions (n = 34,120; mean age at baseline: 46.0 (SD: 17.3); women: 58.1%)	.	Past-month prevalence of binge drinking	.	Critical (2/1/1)
Freeman 2011^40^^,^^b^	USA	1970–2007	Longitudinal, aggregated data; panel data analysis of the state-level beer shipments (provided by the United States Brewers' Association)	.	Level of beer use (gallons of beer per capita)	.	Moderate (3/1/3)
Nelson et al. 2008^41^	USA	1999–2003	Repeated cross-sectional data analysis of the National Survey on Drug Use and Health (n: not reported; age: 12+; women: not reported)	.	Past-month prevalence of alcohol use and binge drinking	.	Critical (0/1/1)
Stehr et al. 2007^42^^,^^b^	USA	1990–2004	Longitudinal, aggregated data; panel data analysis of data from the Distilled Spirits Council of the United States and state statutes	.	Level of alcohol use (annual beverage-specific per capita alcohol sales)	.	Moderate (2/1/3)
Subbaraman et al. 2020^43^^,^^b^	USA	2000–2013	Mixed study design: (a) Longitudinal, aggregated data; per capita consumption data based on the Beverage Information Group Beer, Wine, and Liquor Handbooks, and the National Alcohol Beverage Control Association; and (b) repeated cross-sectional analysis of the National Alcohol Survey (n = 28,251; age: 18+; women: 57.4%)	.	Level of alcohol use (beverage-specific alcohol use per month), past-year alcohol use prevalence	Race/ethnicity	(a): Moderate (2/1/3); (b): Critical (2/1/1)
Gehrsitz et al. 2021^44^^,^^b^	USA (Illinois)	2007–2011	Longitudinal, aggregated data; difference-in-difference models using NielsonIQ Retail Scanner data	90% increase in wine and spirits tax, minor tax increase for beer tax in 2009	Immediate effect on weekly gallons of beer sales	.	Moderate (3/1/3)
Saffer et al. 2022^45^	USA (Illinois)	2007–2011	Longitudinal, aggregated data; synthetic control models using Nielson Homescan data	90% increase in wine and spirits tax, minor tax increase for beer tax in 2009	Immediate effect on monthly per capita alcohol purchases	Income	Moderate (2/1/2)
**Minimum unit pricing**							
Taylor et al. 2021^46^^,^^b^	Australia (Darwin and Palmerston area)	2013–2019	Longitudinal, aggregated data; interrupted time series analysis of alcohol sales data	Introduction of minimum price of $1.30 per standard drink in 2018	Immediate effect on quarterly beverage-specific per capita alcohol consumption	.	Moderate (2/1/3)
O'Brien et al. 2021^47^	Australia (Northern Territory)	2016–2020	Repeated cross-sectional samples of wastewater to obtain the concentration of ethyl sulphate	Introduction of minimum price of $1.30 per standard drink in 2018	Immediate and long-term effect on daily number of standard drinks per 1000 people	.	Moderate (2/1/3)
Stockwell et al., 2012a^48^	Canada (British Columbia)	1989–2010	Longitudinal, aggregated data; time series analysis of quarterly beverage-specific per capita sales data	.	Immediate effect on beverage-specific per capita consumption	.	Moderate (2/1/3)
Stockwell et al., 2012b^49^	Canada (Saskatchewan)	2008–2012	Longitudinal, aggregated data; time series analysis of monthly beverage-specific per capita sales data	Increasing the minimum prices per standard drink for different alcoholic beverages in 2010	Immediate effect on beverage-specific per capita consumption	.	Critical (2/0/3)
Anderson et al. 2021^50^^,^^b^	United Kingdom (Scotland, Wales)	2015–2020	Longitudinal household panel; controlled interrupted time series analysis of the Kantar WorldPanel's household shopping panel^d^ (n = 35,242 households; age: 18+)	Introduction of minimum price of 50 British pence per standard drink in 2018 and 2020	Immediate and long-term effect on daily grams of alcohol purchased per adult per household	.	Critical (2/1/1)
Llopis et al. 2021^51^	United Kingdom (Scotland, Wales)	2015–2020	Longitudinal household panel; controlled interrupted time series analysis of the Kantar WorldPanel's household shopping panel^d^ (n = 70,303 households; age: 18+)	Introduction of minimum price of 50 British pence per standard drink in 2018 and 2020	Immediate and long-term effect on daily grams of beer purchased per adult per household	Income	Critical (2/1/1)
O'Donnell et al. 2019^52^	United Kingdom (Scotland)	2015–2018	Longitudinal household panel; controlled interrupted time series analysis of the Kantar WorldPanel's household shopping panel^d^ (n = 60,132 households; age: 18+)	Introduction of minimum price of 50 British pence per standard drink in 2018	Immediate effect on weekly grams of alcohol purchased per adult per household	Income	Critical (2/1/1)
Rehm et al., 2022b^53^	United Kingdom (Scotland)	2015–2018	Longitudinal household panel; controlled interrupted time series analysis of the Kantar WorldPanel's household shopping panel^d^ (n = 106,490; age: 18+; women: 50.1%)	Introduction of minimum price of 50 British pence per standard drink in 2018	Immediate effect on weekly grams of alcohol purchased per adult per household	Gender, occupation, deprivation	Critical (2/1/1)
Stevely et al. 2022^54^	United Kingdom (Scotland)	2009–2020	Repeated cross-sectional data of the Alcovision cross-sectional panel of past-year alcohol users used in controlled interrupted time series analysis (n = 110,361; age: 18+; women: not reported)	Introduction of minimum price of 50 British pence per standard drink in 2018	Immediate effect on prevalence of monthly harmful alcohol use, number of standard drinks per occasion	Occupation	Critical (3/1/1)
Xhurxhi et al. 2020^55^^,^^b^	United Kingdom (Scotland)	2011–2019	Longitudinal, aggregated data; difference-in-difference models using alcohol sales data from Nielsen	Introduction of minimum price of 50 British pence per standard drink in 2018	Immediate effect on annual on-premises alcohol sales	.	Moderate (3/1/3)
**Temporal availability – hours of sale**							
Rehm et al., 2022a^17^	Estonia, Latvia, Lithuania, Poland	2001–2020	Longitudinal, aggregated data; time-series analysis using total per capita consumption data from the World Health Organization	Reductions in retail hours and night time sales ban for off-premise alcohol sales	Immediate effect on per capita consumption	.	Moderate (2/1/3)
Kolosnitsyna et al. 2014^56^	Russia	2009–2010	Cross-sectional data analysis of monthly alcohol users using the Russian Longitudinal Monitoring Survey (n = 7286; age: not reported; women: 55.1%)	Restricting hours of alcohol sales in 2010	Immediate effect on monthly per capita alcohol consumption	.	Critical (3/0/1)
Bassols et al. 2018^57^	Spain	1998–2004	Repeated cross-sectional data analysis of the Spanish Family Expenditure Survey (n = 17,763; age/women: not reported) and Spanish National Household Survey (n = 78,570; age: not reported; women: 44.9%)	Restricting bar opening hours to close by 2–3.30 am between 1994 and 2011	Immediate effect on annual alcohol expenditures in bars, prevalence of daily wine consumption	Gender	Critical (1/1/1)
Hough et al. 2008^58^	United Kingdom	2004–2006	Repeated cross-sectional; descriptive presentation of alcohol consumption data over time	New alcohol licensing permitting 24-h sales introduced in 2005	Immediate effect on alcohol use (different indicators)	.	n. a.
**Temporal availability – days of sale**							
Carpenter et al. 2009^59^^,^^b^	Canada (Ontario)	1994–1999	Repeated cross-sectional data analysis using the National Population Health Surveys Canada (n = 95,970; age: 20+; women: 51.0%)	Permitting Sunday sales in 1997	Immediate effect on day-specific alcohol consumption	.	Critical (2/1/1)
Grönqvist et al. 2014^60^	Sweden (multiple counties)	1998–2001	Longitudinal, aggregated data; panel data analysis using the alcohol sales data from the state alcohol monopoly	Permitting Saturday sales in 2000	Immediate effect on day-specific alcohol consumption	.	Moderate (3/1/3)
Norström et al. 2005^61^^,^^b^	Sweden (multiple counties)	1995–2002	Longitudinal, aggregated data; controlled interrupted time series analysis using alcohol sales data from the state alcohol monopoly	Permitting Saturday sales in 2000–2001	Immediate and long-term effects on monthly per capita alcohol sales	.	Critical (3/0/3)
Nelson et al. 2008^41^	USA	1999–2003	Repeated cross-sectional data analysis of the National Survey on Drug Use and Health (n: not reported; age: 12+; women: not reported)	Sunday sales ban in 1999–2003	Past-month prevalence of alcohol use and binge drinking	.	Critical (0/1/1)
Stehr et al. 2007^42^^,^^b^	USA	1990–2004	Longitudinal, aggregated data; panel data analysis of data from the Distilled Spirits Council of the United States	Sunday sales ban in 1990–2004	Annual beverage-specific per capita alcohol sales	.	Moderate (3/1/3)
Yörük et al. 2013^62^^,^^b^	USA	1990–2007	Longitudinal, aggregated data; difference-in-difference models using state-level per capita consumption data from the National Institute on Alcohol Abuse and Alcoholism	Permitting Sunday sales in 1990–2007	Annual per capita gallons of alcohol^e^	.	Low (3/2/3)

RTD: ready-to-drink. n. a.: not applicable. USA: United States of America.

a Adopted version of the Newcastle–Ottawa Scale, final score reflects the lowest score across the categories, with a low, moderate and critical risk of bias based on values of 3, 2 and ≤ 1 for the evaluation and outcome category and 2, 1 and 0 for the comparability category, respectively.

b Included in quantitative data summary.

c Only beer tax changes in 2000 and 2001 were included in the analysis, as the 2002 intervention concerned mostly low-strength beer.

d Longitudinal panel in which drop-outs are replaced by new households.

e Alcohol consumption data obtained from the National Institute on Alcohol Abuse and Alcoholism may include industry data used in Stehr et al., 2007 (Distilled Spirits Council of the United States).

### Risk of bias assessment

Most studies had a critical (*n* = 21) or moderate ROB (*n* = 11), with only one study achieving a low ROB in all three categories (see Table 1). A critical ROB was usually linked to the absence of a control group, a missing verification of the implementation of the intended policy, and/or insufficient validity when using individual-level alcohol consumption data.

### Policy 1: alcohol taxation

The majority of tax interventions included in our systematic review reflect increases in alcohol excise taxes, including six studies–all located in the US–that provide tax elasticities.^38^, ^39^, ^40^, ^41^, ^42^, ^44^ Tax reforms were either specific to certain alcoholic beverages such as the beer and ready-to-drink (RTD) tax increases in Australia,^28^, ^29^, ^30^, ^31^ or more complex by affecting multiple alcoholic beverages to varying degrees, such as in Illinois (US),^44^ Lithuania,^33^ or Thailand.^37^

Four studies located in Australia,^31^ Hong Kong,^32^ and the US^42^^,^^44^ evaluated the impact of tax changes on beverage prices, which is the prerequisite for taxes to affect consumption. Specifically, excise taxes levied on alcohol are intended to increase beverage prices, thereby lowering their affordability and consumption. Based on the identified studies, however, taxes were usually over- or under-shifted. For example, following the 2008 tax cut on beer and wine in Hong Kong, the price for wine dropped by 14.3%, while beer prices decreased only marginally.^32^ Similarly, no considerable change in beer prices were observed following the 2000/2001 Australian beer tax reforms.^31^ In the US, an over-shifting of wine and spirits taxes were observed (i.e., prices increased above the tax increase), while beer taxes were under-shifted (i.e., prices did not increase to the same extent as the tax increase).^42^^,^^44^

#### Quantitative summary

**Fig. 2:**
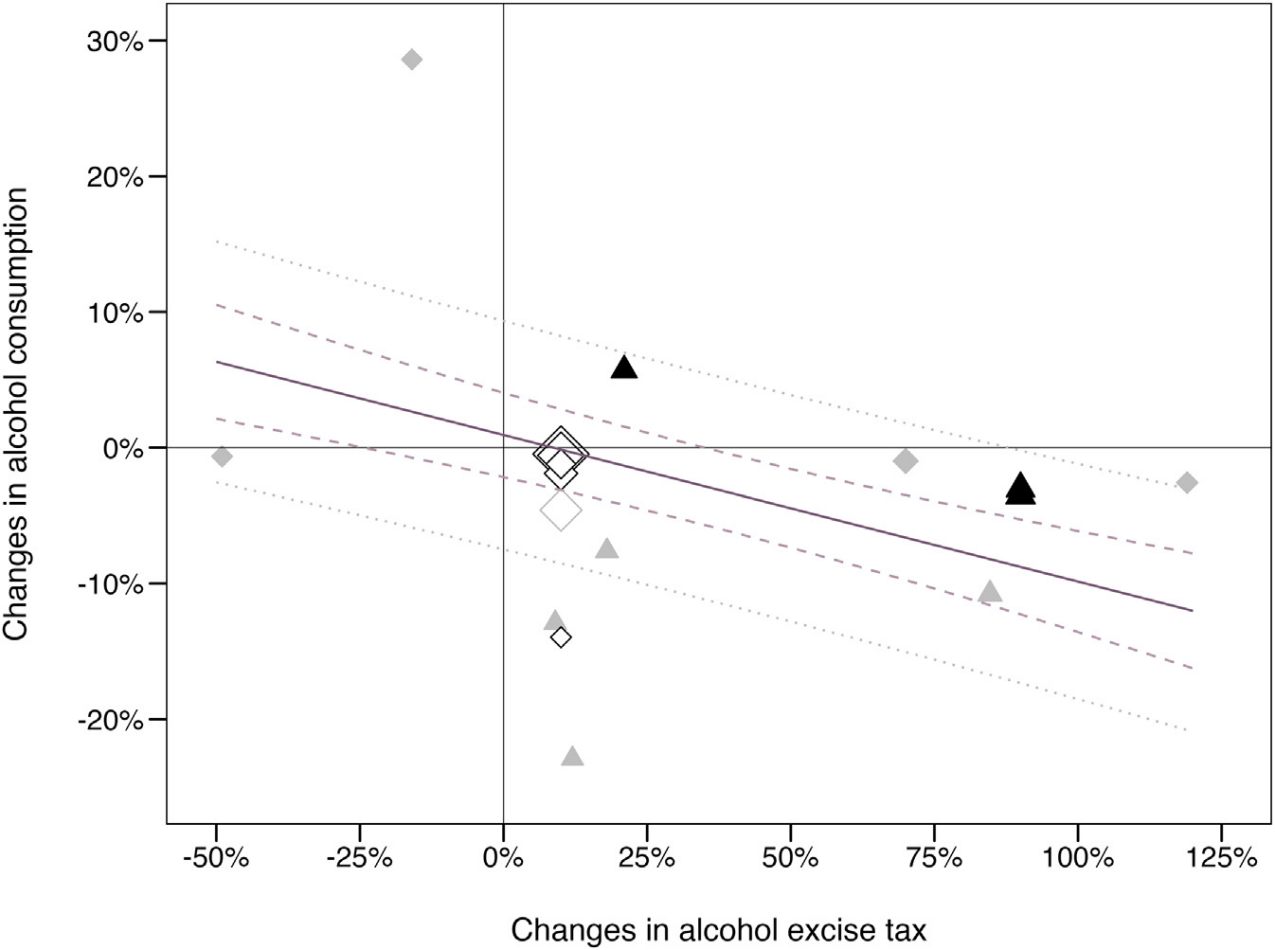
Effect of alcohol excise taxes on the level of alcohol consumption based on the identified literature (*n* = 10). Notes: 1. One study may have provided multiple point estimates, for example, for different alcoholic beverages. 2. Solid shapes indicate the focus of the interventional study: tax reform specific to certain alcoholic beverages (diamond) and a more complex tax reform affecting different alcoholic beverages to varying degrees (triangle). 3. Empty shapes indicate studies reporting tax elasticities. 4. Grey colors indicate studies with a critical risk of bias. 5. The size of the shape indicates the inverse variance weight of each point estimate. 6. The solid regression line depicts the association between alcohol excise taxes and consumption based on the random-effects meta-regression model. The dashed lines illustrate the 95% confidence interval and the dotted grey lines the 95% prediction interval. 7. Where multiple point estimates (k ≥ 2) on the consumption change were available, point estimates were pooled using fixed-effects meta-analysis (applied to one study^28^).

Fig. 2 depicts the association of tax changes and consumption based on studies included in the quantitative summary (*n* = 10 studies). Alcohol tax changes were inversely associated with consumption, suggesting an average reduction in alcohol consumption of 10.8% within the first year of a 100% tax increase (95% confidence interval [CI]: −14.5%, −7.1%, *p* < .001, 95% prediction interval [PI]: −18.5%, −1.2%). This finding was robust against the exclusion of studies on tax decreases, studies with a critical risk of bias, and the one study using a repeated cross-sectional study design; and did not considerably differ from the one-level model (see appendix p. 19). There was substantial heterogeneity between studies (*I*^2^ = 98.4%; Q = 253.84, *p* < .001) that remained unexplained, and no indication for a publication bias given symmetry of the funnel plot (see appendix p. 25). Accounting for the assessment of alcohol use and the policy design (i.e., whether the tax policy concerned a single or multiple beverage categories) did not alter the overall result (appendix p. 19).

#### Qualitative summary

One additional study assessed the immediate impact of a number of tax *increases* on alcohol consumption between 2000 and 2020 in Estonia, Latvia, Lithuania, and Poland using time-series analysis. These tax increases led to a significant reduction in per capita consumption of 0.89 L (95% CI: −1.35, −0.42; data not published but provided by the authors). While this study was not included in the meta-analysis given the range of tax reforms implemented across countries and years, the 2017 Lithuanian tax reform was accounted for by another study.^33^

Very few studies (n = 3) assessed long-term changes in alcohol consumption following tax policy reforms. For tax increases, there was limited evidence that changes in RTD consumption persisted up to nine years after the RTD tax was increased in Australia in 2009.^28^ For tax cuts, however, persistent consumption changes were not observed.^31^^,^^34^ In Russia, for example, where the real term tax dropped by almost 50% following hyperinflation in 1998 and 1999, vodka and liquor retail sales increased markedly in the same years, but declined in subsequent years, although tax levels remained low.^34^

Cross-beverage effects play an important role in the evaluation of tax effectiveness, as there is a risk that some drinkers may shift their consumption to other beverages, if taxes are not raised for all alcoholic beverages. Such cross-beverage effects were indeed observed in those countries where taxes were implemented just for some beverages. For example, while the consumption of RTDs decreased shortly after the RTD tax increase in Australia, the consumption of ciders and spirits increased.^29^^,^^30^ Likewise, the marginal beer tax increase in Illinois, compared to the much higher tax increases on wine and spirits, led to a rise in beer sales by 5.5%.^44^

#### Differences in policy effects across sociodemographic groups

Research on the effectiveness of alcohol taxation conditional on sociodemographics was scarce. Among men, but not among women who had at least six alcoholic drinks in the past six months, spirits consumption increased significantly following reductions in the spirits tax in Switzerland.^35^ This gender difference was, however, not supported by another Swiss study including less frequent drinkers and alcohol abstainers.^36^ With regard to SES, there was some indication that tax changes affected low-income alcohol users to a greater extent,^45^ but this pattern was not observed when education or occupation was used as SES indicator.^36^ Results across racial and ethnic groups were mixed, pointing to some divergent tax elasticities. For example, an increase in alcohol taxes was associated with a significant decrease in consumption levels and past-month prevalence of alcohol use among non-Hispanic Black women and Hispanic women and men in one study,^43^ while another study identified Hispanics to be least responsive to tax changes.^38^

### Policy 2: minimum unit pricing

MUP was recently introduced in Scotland and Wales (UK, Int$ 0.88 per 10 g of pure alcohol),^50^, ^51^, ^52^, ^53^, ^54^, ^55^ and the Northern Territory (Australia, Int$ 0.91 per 10 g of pure alcohol).^46^^,^^47^ In the Canadian provinces British Columbia (BC) and Saskatchewan (SK) MUPs were already in place and increased over the study period.^48^^,^^49^ As there was no fixed price per unit alcohol before the introduction of a MUP, we report the results of studies on the introduction and the increase of MUPs separately. There was no study evaluating MP policies that met our inclusion criteria.

#### Quantitative summary

**Fig. 3:**
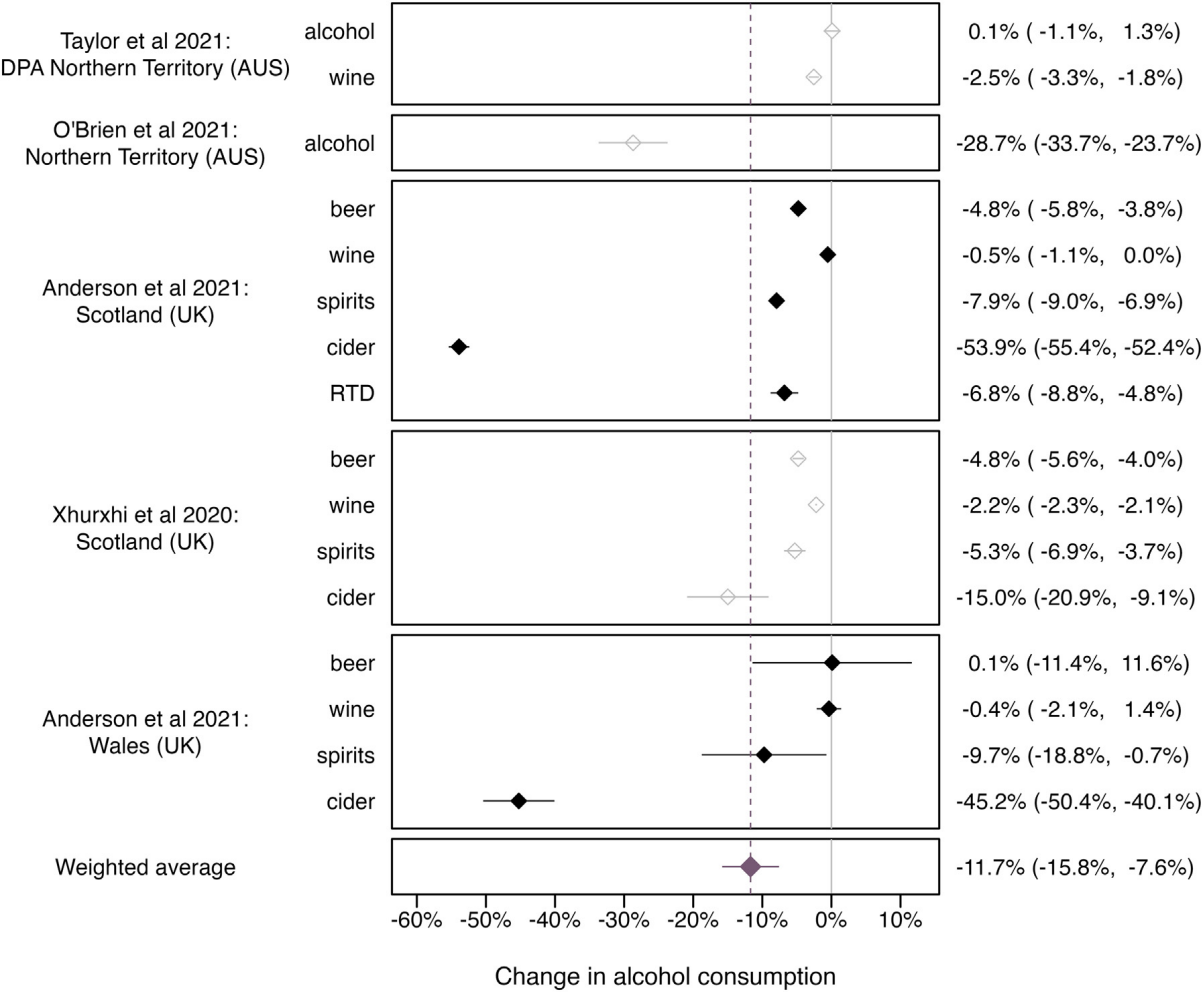
Effect of introducing a minimum unit price on the level of alcohol consumption within one year of policy implementation. 95% prediction interval of weighted average: 95% PI: −28.2%, 5.8%. Notes: 1. Solid and empty shapes indicate individual-level and aggregated alcohol consumption data, respectively. 2. Grey colors indicate studies with a critical risk of bias. 3. DPA = Darwin/Palmerston area is a subregion of the Northern Territory. The point estimates from the Darwin/Palmerston area were preferred over that of the Northern Territory, as another policy were implemented shortly before the introduction of MUPs in different areas throughout the Northern Territory (Police Adjunct Licensing Inspectors, PALI: stationing of police officers in front of off-trade alcohol stores). The introduction of PALIs may have amplified the MUP effect in the Northern Territory. 4. “Alcohol” in Taylor et al., 2021^46^ refers to the consumption of alcoholic beverages other than wine. 5. The effect estimate for ready-to-drink (RTD) beverages in Wales (Anderson et al. 2021^50^) could not be included in the model as the effect and standard error was zero. 6. Where multiple point estimates (k ≥ 2) on the consumption change were available, point estimates were pooled using fixed-effects meta-analysis (applied to one study^47^). 7. Inverse variance weighting: larger confidence interval signifies smaller weighting of a point estimate.

Four studies investigated the immediate impact of the introduction of MUP on beverage-specific and overall consumption.^46^^,^^47^^,^^50^^,^^55^ Within a year post intervention, average alcohol consumption decreased by 11.7% (95% CI: −15.8%, −7.6%, *p* < .001, 95% PI: −28.2%, 5.8%; Fig. 3). Two of these studies also looked at consumption changes two years post intervention, suggesting persistence of consumption declines.^47^^,^^50^

Sensitivity analysis using a random-effects meta-regression model without random intercept revealed that the immediate reduction in consumption following the introduction of MUP was driven by a steep decrease in cider- and RTD-specific consumption (−30.3%, 95% CI: −43.6%, −16.9%; *p* < .001; all other *p*'s > .05). Moreover, significant reductions in consumption following the introduction of MUP were only observed when using individual-level consumption data, which all had a critical risk of bias (see appendix p. 22).

There was substantial between-study heterogeneity in the main models (*I*^2^ = 99.6–99.7%) which did not diminish when excluding individual studies (appendix p. 20). Egger's regression-biased test suggested no publication bias (*p* > .05, see also appendix p. 26).

#### Qualitative summary

In the two Canadian provinces, BC and SK, a 10% increase in the MUP was associated with a significant reduction in per capita consumption for beer (BC: −1.5%, SK: −10.6%), wine (BC: −8.9%, SK: −4.6%), and spirits (BC: −6.8%, SK: −5.9%), as well as for overall consumption (BC: −3.4%, SK: −8.4%).

#### Differences in policy effects across sociodemographic groups

In Scotland, decreases in consumption following the MUP policy that were observed in the overall population were found to be driven primarily by women.^53^ Evidence is mixed on whether the impact of MUP differs by SES, while there was no study on the effectiveness conditional on race and ethnicity. In Scotland, decreases in weekly consumption levels appeared to concentrate in lower rather than higher income groups.^52^ However, this discernible pattern was not supported if deprivation based on the geographical area of living and occupation-based social grade were used as SES indicators.^53^^,^^54^ There was also inconsistent evidence on whether MUP led to a shift towards low-strength beer in certain income groups, with one study supporting such a shift in mid-income households in Scotland^50^ but another not.^51^

### Policy 3: temporal availability

Regulations on the temporal availability of alcoholic beverages were amended in nine countries. Hours of off-premise alcohol sales were modified in Estonia, Latvia, and Lithuania,^17^ as well as in the Russian Federation,^56^ while policies regulating on-premise alcohol sales were implemented in Spain^57^ and the UK.^58^ In the Canadian province of Ontario, Sweden, and the US, alcohol sales were either permitted^59^, ^60^, ^61^, ^62^ or banned^42^ on one additional day.

#### Quantitative summary

**Fig. 4:**
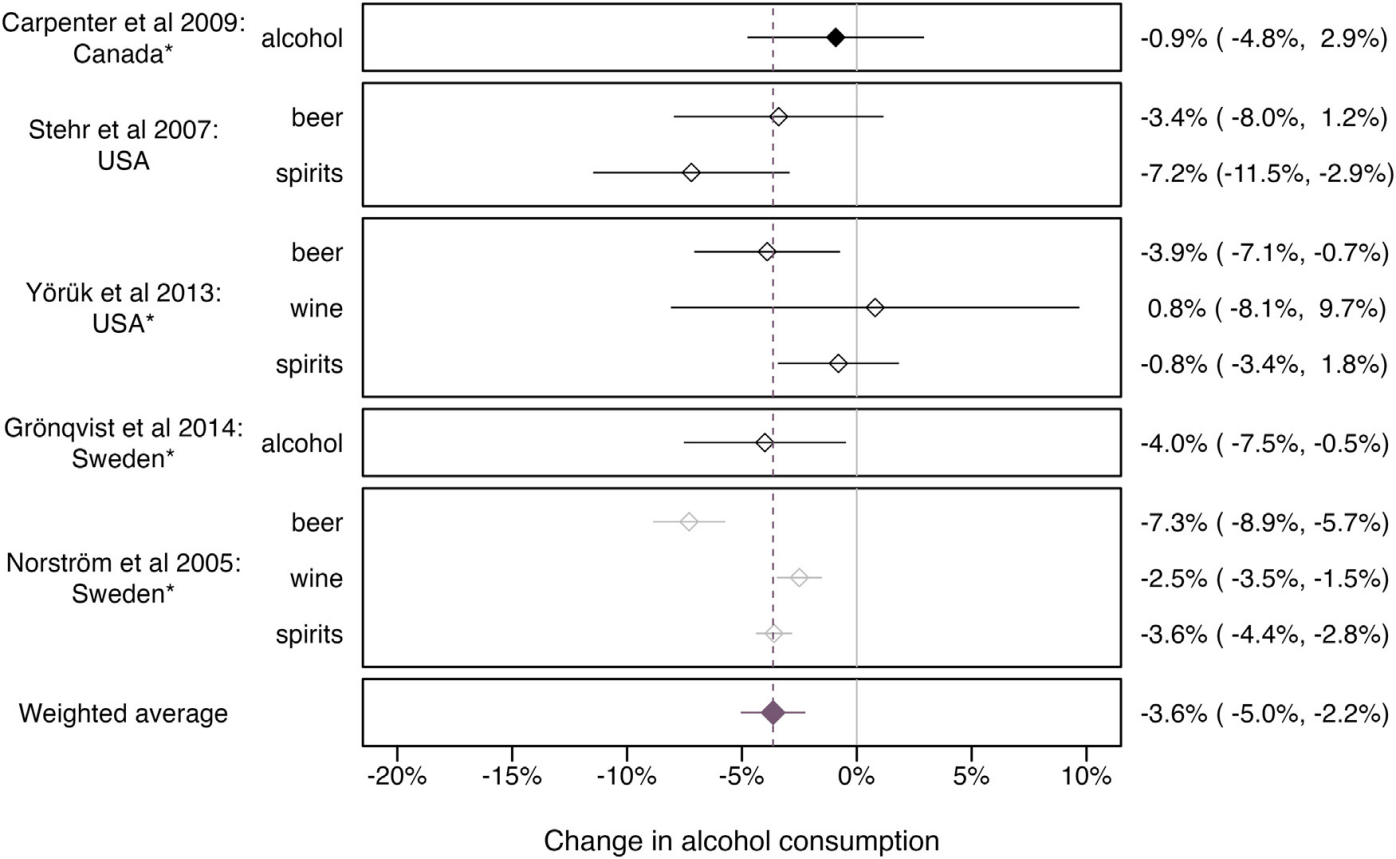
Effect of banning alcohol sales on one additional day on alcohol consumption. 95% prediction interval of weighted average: 95% PI: −7.2%, −0.1%. 1. Solid and empty shapes indicate individual-level and aggregated alcohol consumption data, respectively. 2. Grey colors indicate studies with a critical risk of bias. 3. Asterisks (∗) indicate studies that examined the impact of permitting alcohol sales on one additional day a week; observed consumption changes were inverted. 4. USA = United States of America. 5. Inverse variance weighting; larger confidence interval signifies smaller weighting of a point estimate.

The restriction of alcohol sales by one day led to a 3.6% (95% CI: −5.1%, −2.2%, *p* < .001, 95% PI: −7.2%, −0.1%) decrease in alcohol consumption, according to five studies (Fig. 4). This overall decline was driven by a significant reduction in beer and spirits consumption, whereas the average decrease in wine and overall alcohol consumption was not significantly different from zero (appendix p. 24). Excluding the only repeated cross-sectional study did not alter the overall findings. There was substantial between-study heterogeneity (*I*^2^ = 75.1%), which diminished markedly after excluding one study with a critical risk of bias (*I*^2^ = 31.8%).^61^ Excluding this study, however, did only marginally reduce the overall effect (−3.0%, 95% CI: −4.7%, −1.2%, *p* < .001; see appendix p. 23). There was no indication of publication bias (see appendix p. 27).

#### Qualitative summary

The regulation of on-premise and off-premise alcohol sales hours were also shown to impact alcohol consumption. With regard to on-premise alcohol sales, earlier bar closing hours in Spain resulted in a marked decline in annual average household spending on alcohol, as well as daily and weekly wine consumption in men but not in women.^57^ In England and Wales, where the 2003 Licensing Act allowed 24-h alcohol sales, changes in on-premise sales hours had little impact on consumption^58^; however, it is important to note that most consumption sites have not substantially extended their opening hours following the Licensing Act. For off-premise consumption, some effects on alcohol consumption were found. In the Russian Federation, later closing times of off-premise outlets were linked to higher monthly consumption levels, while a later opening of stores in the morning was associated with lower consumption levels.^56^ Although these restrictions did not include beer, a substitution effect, that is, a shift towards beer drinking, was not identified. In Estonia, Latvia, and Lithuania, night-time alcohol sales bans were not associated with per capita consumption (−0.32 L, 95% CI: −1.07, 0.42, *p* = .390; data not published but provided by the authors).^17^

#### Differences in policy effects across sociodemographic groups

None of the included studies explored the impact of availability restrictions conditional on SES or race and ethnicity.

## Discussion

**Fig. 5:**
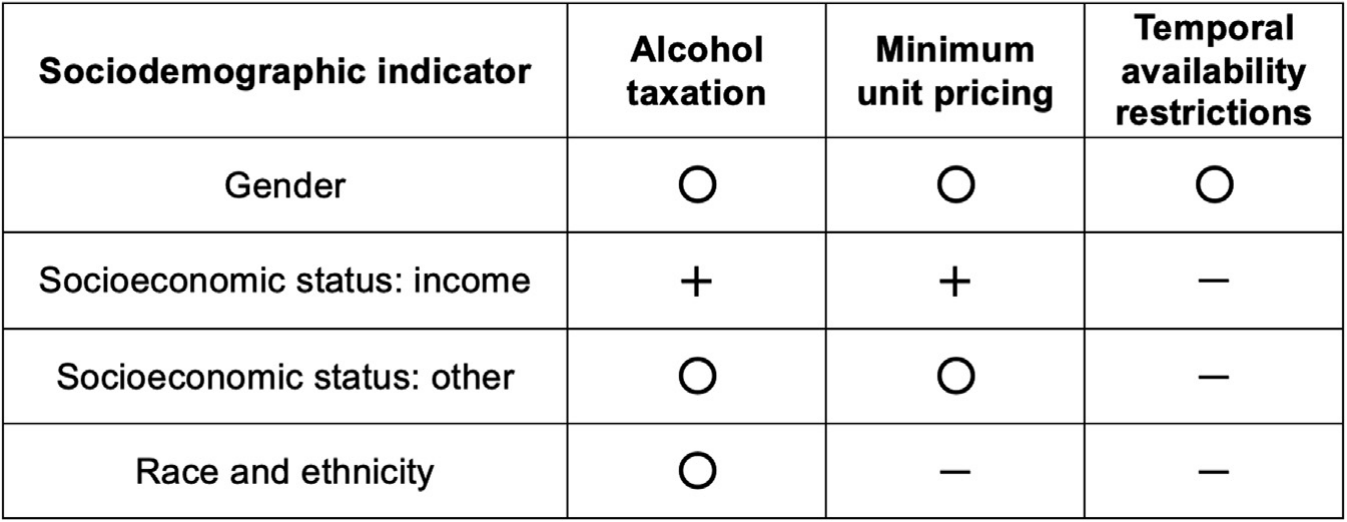
Summary of existing evidence on the impact of alcohol control policies conditional on gender, socioeconomic status, and race and ethnicity. ‘+’ evidence for conditional effectiveness, ‘〇’ mixed or inconclusive evidence for conditional effectiveness, ‘–’ no evidence available.

Our systematic review shows that, consistent with prior research,^8^, ^9^, ^10^ alcohol control policies that raise alcohol prices and reduce the temporal availability of alcoholic beverages lead to a reduction in overall alcohol consumption, with some differential effects identified across sociodemographic groups. Specifically, we found that doubling alcohol excise taxes or introducing a MUP of about Int$ 0.90 per 10 g of pure alcohol results in an average 10% decrease in the level of alcohol consumption within the same year, with potentially larger reductions in low-income compared to more affluent groups. Restricting the temporal availability of alcohol by one day a week also reduces consumption, albeit to a lesser extent than pricing policies. Findings were inconclusive as to whether these policies differentially affect women and men, and there was no study that investigated the differential impact of temporal availability policies by SES or race and ethnicity (see Fig. 5). The sparsity of studies addressing policy effects conditional on gender, SES, and race and ethnicity reveal a major research gap and negligence of such subgroup effects.

In line with prior research, we found pricing policies to be the most promising at lowering consumption levels.^5^, ^6^, ^7^^,^^9^ The reverse linear association of excise taxes and consumption suggest that stronger declines in consumption can be expected with steeper policy-driven price changes, while modest price increases may prove ineffective. This is further supported by sharp declines in cider consumption that were observed in Scotland and Wales following the MUP policy, as the prices of ciders increased most significantly following this policy change. In Scotland, for example, the mean price per unit cider increased by 27% in 2018/2019, while the prices per unit of beer, wine, and spirits increased by around 15%.^55^ At the same time, consumption of alcoholic beverages other than ciders did not increase in these countries, suggesting that there was no shift in consumption, but rather an overall decrease following the introduction of MUP.

When evaluating the impact of these pricing policies, it is worth noting that there is a significant difference between MUP and alcohol taxation. While the MUP raises the floor price of alcoholic beverages, excise taxes will–at least in theory–increase the prices of all alcoholic beverages affected by the policy. However, tax increases do not always translate into higher prices. Prior research on pass-through rates,^63^^,^^64^ as well as the literature presented in this review^31^^,^^32^^,^^42^^,^^44^ demonstrate that taxes are often under- or over-shifted, which may explain some of the observed heterogeneity in our data. With regard to MUP, it is important to bear in mind that the data in this review is based on an absolute threshold in international dollars, based only on the high-income countries Australia, Scotland, and Wales. However, the introduction of this measure is likely to have different impacts depending on the wealth of the countries.

Restrictions on the availability of alcohol, including restricted sales hours and days, were also effective in lowering alcohol consumption (see also^10^). Spontaneous drinking in particular appears to be affected by these restrictions, i.e., drinking on the day or time targeted by the policy. This observation is consistent with declines in injuries and emergency room admissions seen on days where alcohol is less available given sales restrictions.^65^^,^^66^ Moreover, we found some beverage-specific effects that may be related to beverage preferences, reflected by more pronounced decreases in beer and spirits consumption in Canada, Sweden, and the US, while reduced bar opening hours in Spain were linked to a decline of wine drinking.^57^

The second objective of this research was to quantify policy effects conditional on sociodemographic factors, which, however, was not possible given the lack of research on this topic. From the very few studies available, we can conclude that there is a differential effect of pricing policies on consumption across population subgroups. Specifically, the impact of pricing policies varied by income, as seen for alcohol taxation^45^ and MUP.^52^ Such a differential effect was, however, not consistently observed for other SES indicators,^36^^,^^53^^,^^54^ such as occupation, suggesting that the moderating effect observed for income reflects the role of affordability in price-driven consumption reductions. On the other hand, when studying alcohol-related harms, education-based inequalities in all-cause mortality were found to have decreased in Lithuania following the substantial excise tax increases in 2017.^67^ Given the limited number of studies available, further research is needed to elucidate whether the differential policy effects are income-specific or also apply to other SES indicators. For gender and race and ethnicity, research was even more scarce and also inconclusive, preventing any conclusions about differential policy effects.

There are some caveats to consider when drawing conclusions based on our review. First, and most importantly, the evidence presented in this review is confined by a relatively small number of studies, almost exclusively from high-income countries. This limitation may be attributed to the use of English search terms. Second, our study selection criteria led to the exclusion of reports that centred on countries where complex alcohol policy changes happened, such as Finland's 2004 tax reform, which coincidences with new alcohol cross-border purchasing regulations and Estonia's entry to the European Union.^17^^,^^68^ This limitation is, however, also a strength of our research, as this rigorous approach allowed us to study single-policy effectiveness. The majority of included reports relied on longitudinal data, with only two repeated cross-sectional studies included in the quantitative summary. Third, in quantifying the impact of alcohol taxation, we focused on actual tax shifts rather than price elasticities. As discussed earlier, tax changes may not be fully reflected in the beverage price, which we were unable to control for as very few studies reported on real price changes following tax reforms. As previous studies have therefore often looked at price elasticities,^9^ the current study is an important contribution in its focus on tax changes. Fourth, for studies reporting consumption changes among alcohol users only, we assumed that the prevalence of alcohol use remained the same pre and post interventions (see methods section). This assumption was challenged by studies showing that taxation^38^^,^^41^ and availability policies^41^ also affect the prevalence of alcohol use. Given the tendency of the prevalence of alcohol use to decrease rather than to increase with stricter policies, this assumption may have led to an underestimation of the true effect in the overall population. Finally, we have assumed that increasing the restrictiveness of a policy would have an equivalent (inverse) impact to loosening that policy (for sensitivity analyses, see appendix p. 19, 24).

An evidence-informed implementation of alcohol control policies can lead to an immediate reduction in population-level consumption. Such a reduction in consumption may also be sustained in the long-term, presuming that the policies are designed to remain effective over time, which is often not the case.^69^ With lower per capita consumption levels, alcohol-related harms would decrease too, preventing thousands of alcohol-attributable premature deaths globally.^1^^,^^70^ It remains unclear, however, whether different sociodemographic groups benefit equally from these health policies and whether they can help mitigate health inequalities. In light of growing inequalities in the alcohol-attributable health burden,^2^ research on the effectiveness of alcohol policies conditional on sociodemographic factors, including gender, SES, and race and ethnicity, must be prioritised. Moreover, efforts to address alcohol-related health inequalities must be accompanied by upstream policies targeting the root causes of these social inequalities.^16^

## Contributors

Conceptualisation: CK, CP; data curation: CK, TC, LLF; formal analysis: CK; funding acquisition: CP; methodology: CK, CP, YY; project administration: CK; supervision: CP; validation: JML; visualisation: CK; writing – original draft: CK; and writing – review & editing: all authors.

## Data sharing statement

The data file with the information extracted from the individual studies included in the meta-analyses, as well as all R scripts that support the findings of this review are openly available in the Figshare repository at https://doi.org/10.6084/m9.figshare.22593460.v1.

## Declaration of interests

Dr. Kerr has received funding and travel support from the National Alcoholic Beverage Control Association (NABCA). Dr. Kerr has been paid as an expert witness regarding cases on alcohol policy issues retained by the Attorney General's Offices of the US states of Indiana and Illinois under arrangements where half of the cost was paid by organizations representing wine and spirits distributors in those states.

JML has received a PhD stipend from the German Academic Scholarship Foundation.

AL has received Early Postdoc Mobility funding (number P2LAP3 191273) from the Swiss National Science Foundation.

All other authors have no conflicts to declare.
