# Supplementary material for: Reducing alcohol use through alcohol control policies in the general population and population subgroups: a systematic review and meta-analysis
Source: eClinicalMedicine. 2023 May 10;59:101996. doi: 10.1016/j.eclinm.2023.101996 (PMC10225668; doi:10.1016/j.eclinm.2023.101996)
Supplement: Supplementary Figs. S1–S3 and Tables S1–S16 [file mmc1.docx]

# Appendix

[Table S1. Changes from the review protocol (CRD42022339791) 2](#_Toc132104490)

[Table S2. Original search terms by database; search conducted on June 20^th^ 2020. 3](#_Toc132104491)

[Table S3. Search terms by database after modification of the search strategy (for details, see preregistration at Prospero CRD42022339791); search conducted on August 12^th^ (Web of Science, Embase, Medline, PsycInfo) and September 26^th^ 2022 (EconLit). 5](#_Toc132104492)

[Table S4. Reviews and meta-analyses whose reference lists were reviewed for eligible research reports. 7](#_Toc132104493)

[Table S5. Documentation of grey literature search. 9](#_Toc132104494)

[Table S6. Adopted version of the Newcastle-Ottawa Quality Assessment Scale for Cohort Studies. 10](#_Toc132104495)

[Supplement S7: Details on the statistical analysis 11](#_Toc132104496)

[Table S8. List of reports excluded in the full-text screening (systematic search only). 12](#_Toc132104497)

[Table S9. Overview of alcohol control policy interventions covered in the systematic review, by country. 15](#_Toc132104498)

[Table S10. Studies not included in this review because of duplicate data 17](#_Toc132104499)

[Supplement S11: Summary of findings on drinking patterns 18](#_Toc132104500)

[Table S12. Results from the multi-level random-effects meta-regression models on relative changes in alcohol consumption following the implementation of alcohol tax policies. 19](#_Toc132104501)

[Table S13. Results from the random-effects meta-analysis on minimum unit pricing. 20](#_Toc132104502)

[Table S14. Sensitivity analysis: relative changes in alcohol consumption following the introduction of minimum unit pricing. 22](#_Toc132104503)

[Table S15. Results from the random-effects meta-analysis on the restrictions of alcohol sales on one day. 23](#_Toc132104504)

[Table S16. Sensitivity analysis: relative changes in alcohol consumption following the restriction of alcohol sales on one day. 24](#_Toc132104505)

[Figure S1. Funnel plot for the meta-regression model on the impact of alcohol tax changes on relative alcohol consumption changes. 25](#_Toc132104506)

[Figure S2. Funnel plot for the meta-analysis on introducing MUP, impact on alcohol consumption within one year. 26](#_Toc132104507)

[Figure S3. Funnel plot for meta-analysis on the restriction of alcohol sales on one day. 27](#_Toc132104508)

[References 28](#_Toc132104509)

# Table S1. Changes from the review protocol (CRD42022339791)

| **Section** | **Change** |
| --- | --- |
| Searches | An additional systematic literature search was conducted in EconLit via EBSCO. |
| Main outcome(s) | The main outcome was the relative change in alcohol consumption as it provided the most frequently used and comparable outcome. Results on other outcomes, however, are available in the appendix. |
| Risk of bias assessment | The score of the adopted version of the Newcaste-Ottawa Scale ranged between 0 to 8 points. Based on reviewers’ comments, we have applied a different scoring scheme, that is, a critical risk of bias for scores ≤1 for the evaluation and outcome category, and <1 for comparability. |

# Table S2. Original search terms by database; search conducted on June 20^th^ 2020.

| **Web of Science** | TS=(alc* OR beer or wine OR spirit$ OR drink*)  AND TS=(tax* OR minimum pric* OR minimum unit pric* OR ((sale* OR sell* OR) AND hour*))  AND TS=(case-control OR cohort OR time-serie* OR longitudinal OR repeated cross-sectional OR prospective)  NOT ("taxonomy" OR "syntax" OR "excision" OR "taxonomic" OR "taxonomically" OR "taxane" OR "taxi" OR "taxonic" OR parasit* OR microbial OR phenotyp*)  year = “2000-current” |
| --- | --- |
| **Embase/Medline (OVID)** | \| No. \| Searches \| \| --- \| --- \| \| *Alcohol* \| \| \| 1 \| exp Alcohol Drinking/ OR exp *alcoholic beverage/ OR exp *ethanol/ \| \| 2 \| (alc* OR beer or wine OR spirit? OR drink*).ti,ab. \| \| 3 \| 1 or 2 \| \| *Policy measures* \| \| \| 4 \| (tax* OR minimum pric* OR minimum unit pric* ).ti,ab. \| \| 5 \| (((sale* OR sell*) AND hour*)).ti,ab. \| \| 6 \| 4 or 5 \| \| *Study type* \| \| \| 7 \| exp case-control studies/ OR exp cohort studies/ OR exp longitudinal studies/ OR exp prospective studies/ \| \| 8 \| (case-control OR cohort OR time-serie* OR longitudinal OR repeated cross-sectional OR prospective).ti,ab. \| \| 9 \| 7 or 8 \| \| 10 \| 3 and 6 and 9 \| \| *Limitations* \| \| \| 11 \| ("taxonomy" OR "syntax" OR "excision" OR "taxonomic" OR "taxonomically" OR "taxane" OR "taxi" OR "taxonic" OR parasit* OR microbial OR phenotyp*).ti,ab. \| \| 12 \| 10 not 11 \| \| 13 \| limit 12 to yr="2000 -Current" \| |
| **PsycInfo (OVID)** | \| No. \| Searches \| \| --- \| --- \| \| *Alcohol* \| \| \| 1 \| exp Alcohols/ or exp Alcoholic Beverages/ or exp Alcohol Consumption/ \| \| 2 \| (alc* OR beer or wine OR spirit? OR drink*).ti,ab. \| \| 3 \| 1 or 2 \| \| *Policy measures* \| \| \| 4 \| (tax* OR minimum pric* OR minimum unit pric*).ti,ab. \| \| 5 \| (((sale* OR sell*) AND hour*)).ti,ab. \| \| 6 \| 4 or 5 \| \| *Study type* \| \| \| 7 \| exp longitudinal studies/ OR exp prospective studies/ OR exp followup studies/ \| \| 8 \| (case-control OR cohort OR time-serie* OR longitudinal OR repeated cross-sectional OR prospective).ti,ab. \| \| 9 \| 7 or 8 \| \| 10 \| 3 and 6 and 9 \| \| *Limitations* \| \| \| 11 \| ("taxonomy" OR "syntax" OR "excision" OR "taxonomic" OR "taxonomically" OR "taxane" OR "taxi" OR "taxonic" OR parasit* OR microbial OR phenotyp*).ti,ab. \| \| 12 \| 10 not 11 \| \| 13 \| limit 12 to yr="2000 -Current" \| |

# Table S3. Search terms by database after modification of the search strategy (for details, see preregistration at Prospero CRD42022339791); search conducted on August 12^th^ (Web of Science, Embase, Medline, PsycInfo) and September 26^th^ 2022 (EconLit).

| **Web of Science** | TS=(alc* OR beer or wine OR spirit$ OR drink*)  AND TS=(tax* OR minimum pric* OR minimum unit pric* OR price elasticit* OR ((sale* OR sell* OR trad*) AND hour*) OR temporal availability)  AND TS=(case-control OR cohort OR time-serie* OR longitudinal OR repeated cross-sectional OR prospective)  NOT ("taxonomy" OR "syntax" OR "excision" OR "taxonomic" OR "taxonomically" OR "taxane" OR "taxi" OR "taxonic" OR parasit* OR microbial OR phenotyp*)  year = “2000-current” |
| --- | --- |
| **Embase/Medline (OVID)** | \| No. \| Searches \| \| --- \| --- \| \| *Alcohol* \| \| \| 1 \| exp Alcohol Drinking/ OR exp *alcoholic beverage/ OR exp *ethanol/ \| \| 2 \| (alc* OR beer or wine OR spirit? OR drink*).ti,ab. \| \| 3 \| 1 or 2 \| \| *Policy measures* \| \| \| 4 \| (tax* OR minimum pric* OR minimum unit pric* OR price elasticit*).ti,ab. \| \| 5 \| (((sale* OR sell* OR trad*) AND hour*) OR temporal availability).ti,ab. \| \| 6 \| 4 or 5 \| \| *Study type* \| \| \| 7 \| exp case-control studies/ OR exp cohort studies/ OR exp longitudinal studies/ OR exp prospective studies/ \| \| 8 \| (case-control OR cohort OR time-serie* OR longitudinal OR repeated cross-sectional OR prospective).ti,ab. \| \| 9 \| 7 or 8 \| \| 10 \| 3 and 6 and 9 \| \| *Limitations* \| \| \| 11 \| ("taxonomy" OR "syntax" OR "excision" OR "taxonomic" OR "taxonomically" OR "taxane" OR "taxi" OR "taxonic" OR parasit* OR microbial OR phenotyp*).ti,ab. \| \| 12 \| 10 not 11 \| \| 13 \| limit 12 to yr="2000 -Current" \| |
| **PsycInfo (OVID)** | \| No. \| Searches \| \| --- \| --- \| \| *Alcohol* \| \| \| 1 \| exp Alcohols/ or exp Alcoholic Beverages/ or exp Alcohol Consumption/ \| \| 2 \| (alc* OR beer or wine OR spirit? OR drink*).ti,ab. \| \| 3 \| 1 or 2 \| \| *Policy measures* \| \| \| 4 \| (tax* OR minimum pric* OR minimum unit pric* OR price elasticit*).ti,ab. \| \| 5 \| (((sale* OR sell* OR trad*) AND hour*) OR temporal availability).ti,ab. \| \| 6 \| 4 or 5 \| \| *Study type* \| \| \| 7 \| exp longitudinal studies/ OR exp prospective studies/ OR exp followup studies/ \| \| 8 \| (case-control OR cohort OR time-serie* OR longitudinal OR repeated cross-sectional OR prospective).ti,ab. \| \| 9 \| 7 or 8 \| \| 10 \| 3 and 6 and 9 \| \| *Limitations* \| \| \| 11 \| ("taxonomy" OR "syntax" OR "excision" OR "taxonomic" OR "taxonomically" OR "taxane" OR "taxi" OR "taxonic" OR parasit* OR microbial OR phenotyp*).ti,ab. \| \| 12 \| 10 not 11 \| \| 13 \| limit 12 to yr="2000 -Current" \| |
| **EconLit** | (alc* OR beer or wine OR spirit# OR drink*)  AND (tax* OR minimum pric* OR minimum unit pric* OR price elasticit* OR ((sale* OR sell* OR trad*) AND hour*) OR temporal availability)  AND (case-control OR cohort OR time-serie* OR longitudinal OR repeated cross-sectional OR prospective)  NOT ("taxonomy" OR "syntax" OR "excision" OR "taxonomic" OR "taxonomically" OR "taxane" OR "taxi" OR "taxonic" OR parasit* OR microbial OR phenotyp*)  year = “2000-current” |

# Table S4. Reviews and meta-analyses whose reference lists were reviewed for eligible research reports.

| **First author, year** | **Title** | **DOI / PMCID / URL** |
| --- | --- | --- |
| *Alcohol taxation* | | |
| Chaloupka et al 2002 | The Effects of Price on Alcohol Consumption and Alcohol-Related Problems | PMC6683806 |
| Chaloupka et al 2019 | The Use of Excise Taxes to Reduce Tobacco, Alcohol, and Sugary Beverage Consumption | 10.1146/annurev-publhealth-040218-043816 |
| Dhalwani 2011 | A Review of Alcohol Pricing and its Effects on Alcohol Consumption and Alcohol-Related Harm | <https://www.jpmsonline.com/wp-content/uploads/2011/02/JPMS-VOL1-ISSUE1-PAGES23-27-RA.pdf> |
| Elder et al 2010 | The Effectiveness of Tax Policy Interventions for Reducing Excessive Alcohol Consumption and Related Harms | 10.1016/j.amepre.2009.11.005 |
| Gallet et al 2007 | The demand for alcohol: a meta-analysis of elasticities | 10.1111/j.1467-8489.2007.00365.x |
| Nelson 2014 | Gender differences in alcohol demand: a systematic review of the role of prices and taxes | 10.1002/hec.2974 |
| Nelson et al 2013 | Does Heavy Drinking by Adults Respond to Higher Alcohol Prices and Taxes? A Survey and Assessment | 10.1016/S0313-5926(13)50032-4 |
| Nelson et al 2015 | Binge drinking and alcohol prices: a systematic review of age-related results from econometric studies, natural experiments and field studies | 10.1016/j.healthpol.2016.01.018 |
| Nelson et al 2016 | Alcohol prices, taxes, and alcohol-related harms: A critical review of natural experiments in alcohol policy for nine countries | 10.1016/j.healthpol.2016.01.018 |
| Nelson et al 2017 | What happens to drinking when alcohol policy changes? A review of five natural experiments for alcohol taxes, prices, and availability | 10.1002/hec.2974 |
| Patra et al 2012 | Are alcohol prices and taxes an evidence-based approach to reducing alcohol-related harm and promoting public health and safety? A literature review | 10.1177/009145091203900103 |
| Sornpaisarn et al 2013 | Elasticity of alcohol consumption, alcohol-related harms, and drinking initiation in low- and middle-income countries: A systematic review and meta-analysis | 10.7895/ijadr.v2i1.50 |
| Wagenaar et al 2009 | Effects of beverage alcohol price and tax levels on drinking: a meta-analysis of 1003 estimates from 112 studies | 10.1111/j.1360-0443.2008.02438.x |
| Wright et al 2017 | Policy lessons from health taxes: a systematic review of empirical studies | 10.1186/s12889-017-4497-z |
| *Minimum (unit) pricing* | | |
| Boniface et al 2017 | Evidence for the effectiveness of minimum pricing of alcohol: a systematic review and assessment using the Bradford Hill criteria for causality | 10.1136/bmjopen-2016-013497 |
| World Health Organization 2022 | No place for cheap alcohol: the potential value of minimum pricing for protecting lives | <https://www.who.int/europe/publications/i/item/9789289058094> |
| *Temporal availability* | | |
| Gruenewald et al 2011 | Regulating availability: How access to alcohol affects drinking and problems in youth and adults | PMC3860569 |
| Hahn et al 2010 | Effectiveness of policies restricting hours of alcohol sales in preventing excessive alcohol consumption and related harms | 10.1016/j.amepre.2010.09.016 |
| Holmes et al 2014 | The impact of spatial and temporal availability of alcohol on its consumption and related harms: A critical review in the context of UK licensing policies | 10.1111/dar.12191 |
| Middleton et al 2010 | Effectiveness of Policies Maintaining or Restricting Days of Alcohol Sales on Excessive Alcohol Consumption and Related Harms | 10.1016/j.amepre.2010.09.015 |
| Popova et al 2009 | Hours and Days of Sale and Density of Alcohol Outlets: Impacts on Alcohol Consumption and Damage: A Systematic Review | 10.1093/alcalc/agp054 |
| Sanchez-Ramirez et al 2017 | The impact of policies regulating alcohol trading hours and days on specific alcohol-related harms: a systematic review | 10.1136/injuryprev-2016-042285 |
| Sanchez-Ramirez et al 2018 | The impact of policies regulating alcohol trading hours and days on specific alcohol-related harms: a systematic review | 10.1136/injuryprev-2016-042285 |
| Sherk et al 2018 | Alcohol consumption and the physical availability of take-away alcohol: Systematic reviews and meta-analyses of the days and hours of sale and outlet density | 10.15288/jsad.2018.79.58 |
| Stockwell et al 2009 | Do relaxed trading hours for bars and clubs mean more relaxed drinking ? A review of international research on the impacts of changes to permitted hours of drinking | 10.1057/cpcs.2009.11 |
| Wilkinson et al2016 | Impacts of changes to trading hours of liquor licences on alcohol-related harm: a systematic review 2005–2015 | 10.17061/phrp2641644 |
| *Multiple alcohol control policies* | | |
| Anderson et al 2009 | Effectiveness and cost-effectiveness of policies and programmes to reduce the harm caused by alcohol | 10.1016/S0140-6736(09)60744-3 |
| Booth et al 2008 | INDEPENDENT REVIEW OF THE EFFECTS OF ALCOHOL PRICING AND PROMOTION Part A: Systematic Reviews | <https://www.drugsandalcohol.ie/11600/1/DH_091366.pdf> |
| Burton et al 2017 | A rapid evidence review of the effectiveness and cost-effectiveness of alcohol control policies: an English perspective | 10.1016/S0140-6736(16)32420-5 |
| Cook et al 2002 | The Economics Of Alcohol Abuse And Alcohol-Control Policies | 10.1377/hlthaff.21.2.120 |
| Jackson et al 2010 | Interventions on Control of Alcohol Price, Promotion and Availability for Prevention of Alcohol Use Disorders in Adults and Young People | <https://www.nice.org.uk/guidance/ph24/evidence/interventions-on-control-of-alcohol-price-promotion-and-availability-for-prevention-of-alcohol-use-disorders-pdf-371568349> |
| Li et al 2015 | Health promotion interventions and policies addressing excessive alcohol use: a systematic review of national and global evidence as a guide tohealth-care reform in China | 10.1111/add.12784 |
| Martineau et al 2013 | Population-level interventions to reduce alcohol-related harm: An overview of systematic reviews | 10.1016/j.ypmed.2013.06.019 |
| Neufeld et al 2020 | Alcohol control policies in Former Soviet Union countries: A narrative review of three decades of policy changes and their apparent effects | 10.1111/dar.13204 |
| Siegfried et al 2019 | Do alcohol control policies work? An umbrella review and quality assessment of systematic reviews of alcohol control interventions (2006 –2017) | 10.1371/journal.pone.0214865 |
| Wilson et al 2014 | Alcohol interventions, alcohol policy and intimate partner violence: a systematic review | 10.1186/1471-2458-14-881 |

# Table S5. Documentation of grey literature search.

| **Procedure** | The online databases Google and Google Scholar will be searched using the search terms listed below. The first three result pages of each search will be screened for potentially relevant literature that is any publication related to the effectiveness of alcohol policies. If review studies are identified, their reference lists are screened for eligible primary research reports.  The search will be performed in English. |
| --- | --- |
| **Search terms** | - alcohol taxation AND alcohol use - alcohol taxation AND drinking - alcohol taxes AND alcohol use - alcohol taxes AND drinking - excise duties AND alcohol use - excise duties AND drinking - minimum pricing AND alcohol use - minimum pricing AND drinking - minimum unit pricing AND alcohol use - minimum unit pricing AND drinking - temporal availability AND alcohol use - temporal availability AND drinking - sale hours AND alcohol use - sale hours AND drinking - trading hours AND alcohol use - trading hours AND drinking - selling hours AND alcohol use - selling hours AND drinking |
| **Inclusion criteria** | Studies meeting the following criteria will be included: (1) Original research reports of intervention or observational studies (case-control, cohort, or repeated cross-sectional studies) (2) investigating the effectiveness of alcohol control policies (alcohol taxation, MP/MUP, restriction of on-/off-premise sale hours) on (3) individual-level or population-level alcohol consumption (including changes in drinking patterns) (4) compared to a baseline/reference scenario (5)within the population as a whole or within subgroups of interest (i.e. SES, race/ethnicity and sex). |
| **Results** | Any studies or reports of interest will be noted in a separate Excel sheet. The ones available will be marked green, the ones not available online will be marked red and possibly searched for by consulting national experts. |

# Table S6. Adopted version of the Newcastle-Ottawa Quality Assessment Scale for Cohort Studies.

| **Selection (up to 3 points)** | |
| --- | --- |
| **#1 Representativeness of the exposed cohort** | 1. truly representative of the average adult general population in the community (1 point) 2. somewhat representative of the average adult general population in the community (*0* *point*) 3. selected group of users, e.g., nurses, volunteers (0 point) 4. no description of the derivation of the cohort (0 point) |
| **#2 *Was there a control group (e.g., state or country without the policy)?*** | 1. Yes (1 point) 2. No (0 point) 3. No description of a control scenario (0 point) |
| **#3 *Was the implementation of the intended policy ascertained?*** | 1. Yes (1 point) 2. No (0 point) 3. No description (0 point) |
| **Comparability (up to 2 points)** | |
| **#4 Study controls for *year^1^ and state-level effects*** | 1. Yes (1 point) 2. No (0 point) |
| **#5 Study controls for additional factors, *i.e., sex and age; and income (for taxation/MUP studies only)*** | 1. Yes (1 point) 2. No (0 point) |
| **Outcome (up to 3 points)** | |
| **#6 Assessment of alcohol use** | 1. *Recorded per capita consumption, alcohol sales or other statistics* (1 point) 2. Self-report (0 point) 3. No description (0 point) |
| **#7 Was follow-up long enough for outcomes to occur?** | 1. Yes, minimum of one month (1 point) 2. No (0 point) |
| **#8 Adequacy of follow up of cohorts** | 1. *Study based on statistics* (1 point) 2. *Cohort study* with subjects lost to follow up unlikely to introduce bias; follow up rate ≥90% or description of those lost (1 point) 3. *Cohort study* with follow up rate <90% and no description of those lost (0 point) 4. No description *or repeated cross-sectional study* (0 point) |

Note: Modifications to the original scale are in italics. ^1^ i.e., study controls for time trends in alcohol consumption.

Assessment: The final score reflects the lowest score across categories of assessment, with the individual categories scored from 0 to 3 for selection and outcome and from 0 to 2 for comparability. We distinguished between low, moderate and critical risk of bias based on values of 3, 2 and ≤1 for the evaluation and outcome category and 2, 1 and 0 for the comparability category, respectively.

# Supplement S7: Details on the statistical analysis

Fixed-effects meta-analysis to summarise subgroup-specific estimates

Two reports reported policy effects on consumption for different subgroups groups.^1,2^ In these cases, fixed-effects meta-analyses were computed to summarise multiple estimates within each study. The pooled effect estimates were used in subsequent analysis.

Estimating standard errors

Standard errors were used as source of variance and were estimated if not reported. The following formulas were used to estimate the standard error *SE(β)* based on confidence intervals (CI, formula 1), standard deviation (*SD(β)*, formula 2), *p*-value (*p*, formula 3), and *t*-statistic (*t*, formula 4):

Formula 1: $SE(\beta)=\frac{abs(UCI-LCI)}{1.96*2}$ *for n ≥ 50*

Formula 2: $SE(\beta)=\frac{SD(\beta)}{\sqrt{n}}$

Formula 3: $SE(\beta)=\frac{abs(\beta)}{invnormal(1-\frac{p}{2})}$ *for n ≥ 50*

Formula 4: $SE(\beta)=\frac{abs(\beta)}{abs(t)}$

UCI and LCI denote the upper and lower limits of the CI, respectively. When the SE was estimated based on the CI or *p*-value, a normal distribution was assumed for studies with a sample size of *n* ≥ 50.

If the relative change in consumption (*RC*) was estimated based on the absolute change in consumption (β), the following formulas were applied, dependent on whether the SE of the average pre-intervention consumption (*SE(Y)*) was known (formula 5) or not known (formula 6):

Formula 5: $SE\left( RC \right)= \frac{\sqrt{{SE\left( \beta\right)}^{2}+{SE(Y)}^{2}*{(\frac{\beta}{Y})}^{2}}}{Y}$

Formula 6: $SE(RC)= \frac{SE(\beta)}{Y}$

It should be noted that the correlation between β and the average pre-intervention consumption *Y* is assumed to be zero in formula 5. To estimate *SE(RC)* when *SE(Y)* is not known, a fixed population average pre-intervention consumption was assumed.

# Table S8. List of reports excluded in the full-text screening (systematic search only).

| **List of authors** | **Year** | **Title** | **Journal** | **Exclusion reason** |  |
| --- | --- | --- | --- | --- | --- |
| Bloomfield, K.; Karlsson, T.; Grittner, U. | 2016 | How do drinking cultures change? - or do they?: A provisional model based on evidence of transitions in Denmark | Drugs-Education Prevention and Policy | Wrong research design |  |
| Economou, F.; Kountouri, I.; Panagopoulos, Y.; Skintzi, G.; Tsouma, E. | 2022 | Estimating excise tax revenue elasticity and buoyancy for tobacco products and alcoholic beverages: evidence from Greece | Applied Economics | Wrong topic (not about alcohol policies) |  |
| Fogarty, J.; Voon, D. | 2018 | Alcohol Consumption in the United States: Past, Present, and Future Trends | Journal of Wine Economics | Wrong research design |  |
| Giesbrecht, N.; Wettlaufer, A.; Walker, E.; Ialomiteanu, A.; Stockwell, T. | 2012 | Beer, wine and distilled spirits in Ontario: A comparison of recent policies, regulations and practices | Nordic Studies on Alcohol and Drugs | Wrong research design |  |
| Grummon, A. H.; Roberto, C. A.; Lawman, H. G.; Bleich, S. N.; Yan, J. L.; Mitra, N.; Hua, S. V.; Lowery, C. M.; Peterhans, A.; Gibson, L. A. | 2022 | Purchases of Nontaxed Foods, Beverages, and Alcohol in a Longitudinal Cohort After Implementation of the Philadelphia Beverage Tax | Journal of Nutrition | Wrong topic (not about alcohol policies) |  |
| Jiang, H.; Livingston, M. | 2015 | The Dynamic Effects of Changes in Prices and Affordability on Alcohol Consumption: An Impulse Response Analysis | Alcohol and Alcoholism | Wrong research design |  |
| la Cour, L.; Milhoj, A. | 2009 | The sale of alcohol in Denmark - recent developments and dependencies on prices/taxes | Applied Economics | Wrong research design |  |
| Leifman, H.; Trolldal, B. | 2020 | Price and income elasticities for alcohol in Sri Lanka | International Journal of Alcohol and Drug Research | Wrong research design |  |
| Meng, Y.; Brennan, A.; Purshouse, R.; Hill-McManus, D.; Angus, C.; Holmes, J.; Meier, P. S. | 2014 | Estimation of own and cross price elasticities of alcohol demand in the UK-A pseudo-panel approach using the Living Costs and Food Survey 2001-2009 | Journal of Health Economics | Wrong research design |  |
| Moreno-Aguilar, L. A.; Guerrero-Lopez, C. M.; Colchero, M. A.; Quezada-Sanchez, A. D.; Bautista-Arredondo, S. | 2021 | Price elasticity and income elasticity of beer demand in Mexico | Salud Publica De Mexico | Wrong research design |  |
| Pliakas, T.; Egan, M.; Gibbons, J.; Ashton, C.; Hart, J.; Lock, K. | 2018 | Increasing powers to reject licences to sell alcohol: Impacts on availability, sales and behavioural outcomes from a novel natural experiment evaluation | Preventive Medicine | Wrong alcohol control policy |  |
| Room, R.; Bloomfield, K.; Grittner, U.; Gustafsson, N. K.; Makela, P.; Osterberg, E.; Ramstedt, M.; Rehm, J.; Wicki, M.; Gmel, G. | 2013 | What happened to alcohol consumption and problems in the Nordic countries when alcohol taxes were decreased and borders opened? | International Journal of Alcohol and Drug Research | Wrong alcohol control policy |  |
| Stafstrom, M.; Ostergren, P. O. | 2014 | The Impact of Policy Changes on Consumer Behaviour and Alcohol Consumption in Scania, Sweden 1999-2005 | Alcohol and Alcoholism | Wrong alcohol control policy |  |
| Trolldal, B.; Ponicki, W. | 2005 | Alcohol price elasticities in control and license states in the United States, 1982-99 | Addiction | Wrong research design |  |
| Wall, M.; Casswell, S. | 2013 | Affordability of alcohol as a key driver of alcohol demand in New Zealand: a co-integration analysis | Addiction | Wrong research design |  |
| Zhao, J. H.; Stockwell, T. | 2017 | The impacts of minimum alcohol pricing on alcohol attributable morbidity in regions of British Colombia, Canada with low, medium and high mean family income | Addiction | Wrong outcome |  |
| Aage, H. | 2012 | Alcohol in Greenland 1951-2010: Consumption, mortality, prices | International Journal of Circumpolar Health | Wrong research design |  |
| Adrian, M.; Ferguson, B. S.; Her, M. | 2001 | Can alcohol price policies be used to reduce drunk driving? Evidence from Canada | Substance use and misuse | Wrong outcome |  |
| Andreasson, S.; Holder, H. D.; Norstrom, T.; Osterberg, E.; Rossow, I. | 2006 | Estimates of harm associated with changes in Swedish alcohol policy: Results from past and present estimates | Addiction | Wrong outcome |  |
| Chikritzhs, T.; Stockwell, T.; Pascal, R. | 2005 | The impact of the Northern Territory's living with alcohol program, 1992-2002: Revisiting the evaluation | Addiction | Wrong outcome |  |
| Chung, R. Y.; Kim, J. H.; Yip, B. H.; Wong, S. Y. S.; Wong, M. C. S.; Chung, V. C. H.; Griffiths, S. M. | 2014 | Alcohol tax policy and related mortality. An age-period-cohort analysis of a rapidly developed Chinese population, 1981-2010 | Plos One | Wrong outcome |  |
| Fairman, B. J.; Simons-Morton, B. G.; Haynie, D. L.; Liu, D.; Goldstein, R. B.; Hingson, R. W.; Gilman, S. E. | 2019 | State alcohol policies, taxes, and availability as predictors of adolescent binge drinking trajectories into early adulthood | Addiction (Abingdon, England) | Wrong study sample |  |
| Falkner, C.; Christie, G.; Zhou, L.; King, J. | 2015 | The effect of alcohol price on dependent drinkers' alcohol consumption | New Zealand Medical Journal | Wrong research design |  |
| Kerr, W. C.; Williams, E.; Ye, Y.; Subbaraman, M. S.; Greenfield, T. K. | 2018 | Survey Estimates of Changes in Alcohol Use Patterns Following the 2012 Privatization of the Washington Liquor Monopoly | Alcohol & Alcoholism | Wrong alcohol control policy |  |
| LeClercq, J.; Bernard, S.; Mucciaccio, F.; Esser, M. B. | 2021 | Prospective Analysis of Minimum Pricing Policies to Reduce Excessive Alcohol Use and Related Harms in U.S. States | Journal of Studies on Alcohol and Drugs | Wrong research design |  |
| Makela, P.; Bloomfield, K.; Gustafsson, N. K.; Huhtanen, P.; Room, R. | 2008 | Changes in volume of drinking after changes in alcohol taxes and travellers' allowances: results from a panel study | Addiction | Wrong alcohol control policy |  |
| Mohler-Kuo, M.; Rehm, J.; Heeb, J. L.; Gmel, G. | 2004 | Decreased Taxation, Spirits Consumption and Alcohol-Related Problems in Switzerland | Journal of Studies on Alcohol | Wrong outcome |  |
| Naimi, T.; Blanchette, J.; Lira, M. C.; Heeren, T.; Smart, R.; Pacula, R. | 2021 | Research on state alcohol and cannabis policy environments: What have we learned? | Alcoholism: Clinical and Experimental Research | Conference abstract |  |
| Razvodovsky, Y. E. | 2013 | Alcohol affordability and epidemiology of alcoholism in Belarus | Alcoholism | Wrong research design |  |
| Sharma, A.; Vandenberg, B. | 2019 | Heterogenous wealth effects of minimum unit price on purchase of alcohol: Evidence using scanner data | Plos One | Wrong research design |  |
| So, V.; Millard, A. D.; Katikireddi, S. V.; Forsyth, R.; Allstaff, S.; Deluca, P.; Drummond, C.; Ford, A.; Eadie, D.; Fitzgerald, N.; Graham, L.; Hilton, S.; Ludbrook, A.; McCartney, G.; Molaodi, O.; Open, M.; Patterson, C.; Perry, S.; Phillips, T.; Schembri, G.; Stead, M.; Wilson, J.; Yap, C.; Bond, L.; Leyland, A. H. | 2021 | Intended and unintended consequences of the implementation of minimum unit pricing of alcohol in Scotland: a natural experiment | NIHR Journals Library. Public Health Research | Wrong study sample |  |
| Chaudhary, S.; MacKey, W.; Duncan, K.; Forrest, E. H. | 2022 | Changes in Hospital Discharges with Alcohol-Related Liver Disease in a Gastroenterology and General Medical Unit Following the Introduction of Minimum Unit Pricing of Alcohol: The GRI Q4 Study | Alcohol & Alcoholism | Wrong study sample |  |
| Chikritzhs, T.; Stockwell, T. | 2002 | The impact of later trading hours for Australian public houses (hotels) on levels of violence | Journal of Studies on Alcohol | Wrong outcome |  |
| Curtis, A.; Coomber, K.; Droste, N.; Hyder, S.; Palmer, D.; Miller, P. G. | 2017 | Effectiveness of community-based interventions for reducing alcohol-related harm in two metropolitan and two regional sites in Victoria, Australia | Drug and Alcohol Review | Wrong outcome |  |
| Goryakin, Y.; Roberts, B.; McKee, M. | 2015 | Price elasticities of alcohol demand: evidence from Russia | European Journal of Health Economics | Wrong research design |  |
| Lin, C. M.; Liao, C. M. | 1544 | Alcohol tax policy in relation to hospitalization from alcohol-attributed diseases in Taiwan: A nationwide population analysis of data from 1996 to 2010 | Alcoholism: Clinical and Experimental Research | Wrong outcome |  |
| Manthey, J.; Jasilionis, D.; Rehm, J.; Stelemekas, M. | 2022 | Socioeconomic Status-Specific Alcohol Policy Effects | Alcoholism: Clinical and Experimental Research | Conference abstract |  |
| Miller, P.; Curtis, A.; Palmer, D.; Busija, L.; Tindall, J.; Droste, N.; Gillham, K.; Coomber, K.; Wiggers, J. | 2014 | Changes in injury-related hospital emergency department presentations associated with the imposition of regulatory versus voluntary licensing conditions on licensed venues in two cities | Drug and Alcohol Review | Wrong outcome |  |
| Bask, M.; Melkersson, M. | 2004 | Rationally addicted to drinking and smoking? | Applied Economics | Wrong research design |  |
| Casswell, S.; Huckle, T.; Wall, M.; Yeh, L. C. | 2014 | International Alcohol Control Study: Pricing Data and Hours of Purchase Predict Heavier Drinking | Alcoholism-Clinical and Experimental Research | Wrong research design |  |
| Grosova, S.; Masar, M.; Kutnohorska, O.; Kubes, V. | 2017 | Demand for Beer in the Czech Republic: Understanding Long-Term On- and Off-Trade Price Elasticities | Czech Journal of Food Sciences | Wrong research design |  |
| Koksal, A.; Wohlgenant, M. | 2016 | Pseudo panel data estimation technique and rational addiction model: an analysis of cigarette, alcohol and coffee demands | Agricultural Economics | Wrong research design |  |
| Livingston, M.; Coomber, K.; de Andrade, D.; Taylor, N.; Ferris, J.; Puljevic, C.; Miller, P. G. | 2021 | Assessing the impact of Queensland's late-night alcohol restrictions using health system data | Drug and Alcohol Review | Wrong outcome |  |
| Mazerolle, L.; White, G.; Ransley, J.; Ferguson, P. | 2012 | Violence in and around Entertainment Districts: A Longitudinal Analysis of the Impact of Late-Night Lockout Legislation | Law & Policy | Wrong outcome |  |
| Sen, A.; Luong, M. | 2008 | Estimating the Impact of Beer Prices on the Incidence of Sexually Transmitted Diseases: Cross-Province and Time Series Evidence from Canada | Contemporary Economic Policy | Wrong outcome |  |
| Staudigel, M.; Schrock, R. | 2015 | Food Demand in Russia: Heterogeneous Consumer Segments over Time | Journal of Agricultural Economics | Wrong research design |  |
| Wong, L.; Selvanathan, E. A.; Selvanathan, S. | 2017 | Empirical analysis of Australian consumption patterns | Empirical Economics | Wrong topic (not about alcohol policies) |  |
| Saffer, H.; Dave, D.; Grossman, M. | 2016 | A Behavioral Economic Model of Alcohol Advertising and Price | Health Economics | Wrong research design |  |
| Pierani, P.; Tiezzi, S. | 2007 | Addiction and Alcohol Consumption: Evidence from Italian Data | Rivista Internazionale di Scienze Sociali | Wrong research design |  |
| Mayo, J. R. | 2000 | An Estimate of U.S. Demand for Alcoholic Beverages, 1986-92 | Pennsylvania Economic Review | Wrong research design |  |
| Gius, M. P. | 2002 | The Effect of Taxes on Alcoholic Consumption: An Individual Level of Analysis with a Correction for Aggregate Public Policy Variables | Pennsylvania Economic Review | Wrong study sample |  |
| Cook, Philip J.; Peters, Bethany | 2005 | The Myth of the Drinker's Bonus | n. a. | Wrong outcome |  |

n. a. = not applicable.

# Table S9. Overview of alcohol control policy interventions covered in the systematic review, by country.

| **Country or territory** | **Intervention** | **Year(s) of intervention** |
| --- | --- | --- |
| **Alcohol taxation**^*^ | | |
| Australia^3^ | 85-154% *increase* in excise tax on low-, mid-, and high-strength beer (2000), 29-64% *decrease* in excise tax on low-, mid-, and high-strength beer sold on-premises (2001) and off-premises (2002; low-strength and some mid-strength beers only) | 2000-2002 |
| Australia^1,4,5^ | 70% *increase* in excise tax on spirits-based ready-to-drink beverages (2008) as well as beer- and wine-based ready-to-drink beverages (2009) | 2008-2009 |
| Estonia^6^ | Excise taxes were *increased* multiple times between 2008 and 2018, and a *reduction* of excise taxes by 20% in 2019^†^ | 2008, 2010, 2016, 2017, 2018, 2019 |
| Hong Kong (China)^7^ | *Removal* of excise taxes on beer and wine | 2008 |
| Illinois (United States)^8,9^ | 90% *increase* in excise taxes on wine and spirits, 21% *increase* in excise tax on beer | 2009 |
| Latvia^6^ | Excise taxes were *increased* in 2009, 2010, and 2019^†^ | 2009, 2010, 2019 |
| Lithuania^6,10^ | Excise taxes were increased in 2008 and 2017^†^; in 2017, there was a 112% *increase* in the excise taxes on beer and wine and 23% *increase* in the excise tax on spirits | 2008, 2017 |
| Poland^6^ | Excise taxes were *increased* in 2009 and 2020^†^ | 2002, 2009, 2020 |
| United States^11–16^ | Various state-specific tax *increases* and *decreases* throughout the period | 1970-2013 |
| Russian Federation^17^ | 47% *decrease* in excise tax on spirits, as excise taxes were not adjusted to hyperinflation | 1998-1999 |
| Switzerland^18–20^ | Introduction of uniform excise tax for domestic and foreign spirits; 9-50% *decrease* in the excise tax for foreign spirits and a 12% *increase* in the excise tax on domestic spirits | 1999 |
| Thailand^21^ | *Increase* in excise tax on various alcoholic beverages by on average 18% (2005), 9% (2007), and 12% (2009) | 2005, 2007, 2009 |
| **Minimum unit pricings** | | |
| British Columbia (Canada)^22^ | Several *increases* of beverage-specific minimum unit prices throughout the period | 1989-2010 |
| Northern Territory (Australia)^2,23,24^ | *Introduction* of a minimum unit price of 1·30 Australian dollar per 10 grams of pure alcohol | 2018 |
| Saskatchewan (Canada)^25^ | *Increases* of minimum unit prices for alcoholic beverages from 1·39 to 1·56 Canadian dollar per 13·5 grams of pure alcohol | 2010 |
| Scotland (United Kingdom)^26–33^ | *Introduction* of a minimum unit price of 50 British pence per 8 grams of pure alcohol | 2018 |
| Wales (United Kingdom)^27,29^ | *Introduction* of a minimum unit price of 50 British pence per 8 grams of pure alcohol | 2020 |
| **Temporal availability – hours of sale** | | |
| Estonia^6^ | *Restriction* of off-premise sales of alcoholic beverages between 10 p.m. and 10 a.m. | 2008 |
| Latvia^6^ | *Restriction* of off-premise sales of alcoholic beverages between 10 p.m. and 8 a.m. | 2002 |
| Lithuania^6^ | *Restriction* of off-premise alcohol sales between 10 p.m. and 8 a.m. (2009), *restriction* of off-premise sales between 8 p.m. and 10 a.m. on Mondays to Saturday and 3 p.m. and 10 a.m. on Sundays (2018) | 2009, 2018 |
| Russian Federation^34^ | *Restriction* of off-premises alcohol sales between 11 p.m. and 8 a.m. within the entire country (2011); some restrictions of off-premises alcohol sales were already introduced in few regions in 2009 and most regions in 2010 | 2009-2011 |
| Spain^35^ | *Restriction* of bar opening hours from 6 a.m. to 2.30-3.00 a.m., introduced at state level at some point between 1994 and 2011 | 1994-2011 |
| United Kingdom^36^ | *Abolishment* of licensing hours for on-premises consumption sites, which theoretically allow for 24-hour sales of alcohol (2003 Licensing Act) | 2005 |
| **Temporal availability – days of sale** | | |
| Ontario (Canada)^37^ | *Permitting* Sunday sales | 1997 |
| Sweden^38–40^ | *Permitting* Saturday sales (selected counties) | 2000-2001 |
| United States^14,15,41^ | *Permitting* or *restricting* Sunday sales in various states | 1990-2007 |

*None of the eligible studies indicated whether the taxation policies were linked to inflation. ^†^It should be noted that Estonia, Latvia, Lithuania, and Poland are Member States of the European Union since May 1^st^, 2004 and applied Schengen area regulations since December 21^st^, 2007, which may have impacted alcohol consumption.

# Table S10. Studies not included in this review because of duplicate data

| **Study included** | **Study not included because of duplicate data** |
| --- | --- |
| Freeman 2011 | Freeman DG. Alternative Panel Estimates of Alcohol Demand, Taxation, and the Business Cycle. South Econ J 2000; 67: 325.  Kubik JD, Moran JR. Can Policy Changes Be Treated as Natural Experiments? Evidence from State Excise Taxes. SSRN Electron J 2002. DOI:10.2139/ssrn.1808888. |
| Heeb et al 2003 | Gmel G, Wicki M, Rehm J, Heeb J-L. Estimating regression to the mean and true effects of an intervention in a four-wave panel study. Addiction 2007; 103: 32–41. |
| Llopis et al 2021 | Anderson P, Kokole D, Jané Llopis E. Impact of minimum unit pricing on shifting purchases from higher to lower strength beers in Scotland: Controlled interrupted time series analyses, 2015–2020. Drug Alcohol Rev 2022; 41: 646–56. |
| Norström et al 2005 | Norström T, Skog O-J. Saturday opening of alcohol retail shops in Sweden: an impact analysis. J Stud Alcohol 2003; 64: 393–401. |
| Taylor et al 2021 | Coomber K, Miller P, Taylor N, et al. Investigating the introduction of the alcohol minimum unit price in the Northern Territory (Final report). Geelong: Deakin University, 2020. |
| Xhurxhi et al 2020 | Giles L, Robinson M, Beeston C. Minimum Unit Pricing (MUP) for alcohol evaluation. Sales-based consumption: a descriptive analysis of one-year post-MUP off-trade alcohol sales data. Edinburgh, UK: NHS Health Scotland, 2019 <http://www.healthscotland.scot/media/2954/c-users-kims-desktop-sales-based-consumption-descriptive-analysis-of-one-year-post-mup-off-trade-alcohol-sales-data.pdf>.  Robinson M, Mackay D, Giles L, Lewsey J, Richardson E, Beeston C. Evaluating the impact of minimum unit pricing (MUP) on off‐trade alcohol sales in Scotland: an interrupted time–series study. Addiction 2021; 116: 2697–707. |

# Supplement S11: Summary of findings on drinking patterns

Alcohol taxation

Tax changes also affected alcohol consumption patterns, although findings were inconclusive, indicating a lower^13^ and higher^14^ prevalence of alcohol use and an unchanged^14^ or lower prevalence of binge drinking following tax increases. In Hong Kong, the elimination of the excise tax on beer and wine resulted in a substantial increase in past-year and lifetime alcohol use three to four years post intervention.^7^

Minimum unit pricing

The impact of MUP on changes in consumption patterns has rarely been studied and was limited to Scotland. Based on the available evidence, MUP appears to have had no impact on the drinking frequency, while the number of units per drinking occasion decreased.^32^ Moreover, a steeper decline in consumption was observed among heavier drinkers, with the exception of those with the highest drinking levels,^28,32^ as well as in households with higher daily alcohol purchases.^26^

Temporal availability

Restricting or permitting alcohol sales on an additional day was shown to impact alcohol consumption patterns, particularly on the day affected by the policy. In the US, banning alcohol sales on Sundays led to an overall decrease in the past-month prevalence of alcohol use and heavy episodic drinking.^14^ Similarly, the permission of Sunday sales in Ontario^37^ and Saturday sales in Sweden^40^ led to an increase in the day-specific prevalence of alcohol use, binge drinking and drinking quantity, and a 10.3% increase in day-specific alcohol use, respectively. The prevalence of past-week alcohol use, however, did not change after Sunday sales were permitted in Ontario.^37^

# Table S12. Results from the multi-level random-effects meta-regression models on relative changes in alcohol consumption following the implementation of alcohol tax policies.

| **Outcome variable: relative change in alcohol consumption** | | | | **Between-study heterogeneity** | |
| --- | --- | --- | --- | --- | --- |
| **Predictor** | **Estimate** | **95% CI** | **p-value** | **I^2^ (%)*** | **Q (p-value)** |
| *Main model (with random intercept, k = 17)* | | | | | |
| Relative change in alcohol excise tax | -0·108 | -0·145, -0·071 | < ·001 | 98·4 | 253·84 (< ·001) |
| Per capita GDP PPP (z-standardized) | 0·036 | -0·000, 0·071 | 0·051 |  |  |
| *Sensitivity analysis 1: main model (with random intercept, k = 17), study identifier included as covariate* | | | | | |
| Relative change in alcohol excise tax | -0·119 | -0·188, -0·050 | 0·007 | 31·7 | 7·32 (·198) |
| Per capita GDP PPP (z-standardized) | -1·071 | -2·633, 0·492 | 0·138 |  |  |
| *Sensitivity analysis 2: including reports on tax increases only (with random intercept, k = 15)* | | | | | |
| Relative change in alcohol excise tax | -0·108 | -0·146, -0·070 | < ·001 | 98·5 | 242·96 (< ·001) |
| Per capita GDP PPP (z-standardized) | 0·034 | -0·001, 0·069 | ·060 |  |  |
| *Sensitivity analysis 3: assessment of alcohol use (with random intercept, k = 17)* | | | | | |
| Relative change in alcohol excise tax | -0·109 | -0·147, -0·072 | < ·001 | 98·9 | 85·36 (<·001) |
| Per capita GDP PPP (z-standardized) | 0·035 | -0·006, 0·077 | ·093 |  |  |
| Individual-level consumption data (ref: aggregated data) | 0·030 | -0·044, 0·105 | ·428 |  |  |
| *Sensitivity analysis 4: policy design (with random intercept, k = 17)* | | | | | |
| Relative change in alcohol excise tax | -0·107 | -0·0145, -0·069 | < ·001 | 98·8 | 248·79 (<·001) |
| Per capita GDP PPP (z-standardized) | 0·037 | -0·004, 0·076 | ·074 |  |  |
| Beverage-specific policy (ref: policy affected multiple beverages) | 0·024 | -0·048, 0·100 | ·516 |  |  |
| *Sensitivity analysis 5: exclude studies with critical risk of bias (with random intercept, k = 8)* | | | | | |
| Relative change in alcohol excise tax | -0·122 | -0·164, -0·080 | < ·001 | 99·1 | 33·89 (< ·001) |
| Per capita GDP PPP (z-standardized) | 0·019 | -0·033, 0·071 | ·474 |  |  |
| *Sensitivity analysis 6: exclude one study with a repeated cross-sectional study design (with random intercept, k = 16)* | | | | | |
| Relative change in alcohol excise tax | -0·110 | -0·147, -0·073 | < ·001 | 98·1 | 66·83 (< ·001) |
| Per capita GDP PPP (z-standardized) | 0·033 | -0·004, 0·071 | ·078 |  |  |

NOS: Newcastle-Ottawa scale for cohort studies to access risk of bias. 95% CI: confidence interval. Ref: reference. *Total variance.

# Table S13. Results from the random-effects meta-analysis on minimum unit pricing.

| **Model** | **Estimate** | **95% CI** | **p-value** | **Between-study heterogeneity** | | **Egger’s regression test for publication bias** |
| --- | --- | --- | --- | --- | --- | --- |
|  |  |  |  | **I^2^** | **Q (p-value)** | **z (p-value)** |
| Main model: consumption change within one year (k = 16) | -0·117 | -0·158, -0·076 | < ·001 | 99·7 | 5249·51 (< ·001) | -1·23 (·219) |
| Leave-one-out sensitivity analysis – consumption change within one year | | | | | | |
| Anderson et al_2021: RTD (Scotland) | -0·120 | -0·16, -0·08 | < ·001 | 99·7 | 5232·51 (< ·001) | · |
| Anderson et al_2021: beer (Scotland) | -0·122 | -0·17, -0·08 | < ·001 | 99·7 | 5231·36 (< ·001) | · |
| Anderson et al_2021: beer (Wales) | -0·122 | -0·16, -0·08 | < ·001 | 99·7 | 5249·30 (< ·001) | · |
| Anderson et al_2021: cider (Scotland) | -0·073 | -0·09, -0·06 | < ·001 | 97·9 | 668·57 (< ·001) | · |
| Anderson et al_2021: cider (Wales) | -0·096 | -0·14, -0·05 | < ·001 | 99·7 | 4986·55 (< ·001) | · |
| Anderson et al_2021: spirits (Scotland) | -0·120 | -0·16, -0·08 | < ·001 | 99·7 | 5144·80 (< ·001) | · |
| Anderson et al_2021: spirits (Wales) | -0·118 | -0·16, -0·08 | < ·001 | 99·7 | 5247·19 (< ·001) | · |
| Anderson et al_2021: wine (Scotland) | -0·125 | -0·18, -0·07 | < ·001 | 99·7 | 5188·13 (< ·001) | · |
| Anderson et al_2021: wine (Wales) | -0·125 | -0·17, -0·08 | < ·001 | 99·7 | 5243·29 (< ·001) | · |
| O'Brien et al_2021: alcohol (Northern Terr | -0·106 | -0·15, -0·06 | < ·001 | 99·7 | 5144·27 (< ·001) | · |
| Taylor et al_2021: alcohol (Darwin/Palmers | -0·125 | -0·17, -0·08 | < ·001 | 99·7 | 5231·00 (< ·001) | · |
| Taylor et al_2021: wine (Darwin/Palmerston | -0·124 | -0·17, -0·08 | < ·001 | 99·7 | 5249·49 (< ·001) | · |
| Xhurxhi et al_2020: beer (Scotland) | -0·122 | -0·17, -0·08 | < ·001 | 99·7 | 5220·61 (< ·001) | · |
| Xhurxhi et al_2020: cider (Scotland) | -0·115 | -0·16, -0·07 | < ·001 | 99·7 | 5232·53 (< ·001) | · |
| Xhurxhi et al_2020: spirits (Scotland) | -0·122 | -0·16, -0·08 | < ·001 | 99·7 | 5237·86 (< ·001) | · |
| Xhurxhi et al_2020: wine (Scotland) | -0·124 | -0·18, -0·06 | < ·001 | 99·7 | 4917·24 (< ·001) | · |

RTD: ready-to-drink.

# Table S**14**. Sensitivity analysis: relative changes in alcohol consumption following the introduction of minimum unit pricing.

| **Outcome variable: relative change in alcohol consumption** | | | | **Between-study heterogeneity** | |
| --- | --- | --- | --- | --- | --- |
| **Predictor** | **Estimate** | **95% CI** | **p-value** | **I^2^ (%)*** | **Q (p-value)** |
| *Model 1: consumption changes within one year* | | | | | |
| *Sensitivity analysis 1 (without intercept): beverage-specific or overall consumption (k = 16)* | | | | | |
| Alcohol consumption (not beverage-specific) | -0·141 | -0·329, 0·048 | ·144 | 99·9 | 1604·77 (< ·001) |
| Beer-specific consumption | -0·033 | -0·190, 0·123 | ·676 |  |  |
| Wine-specific consumption | -0·014 | -0·146, 0·118 | ·834 |  |  |
| Spirits-specific consumption | -0·076 | -0·231, 0·079 | ·338 |  |  |
| Cider- or RTD-specific consumption | -0·303 | -0·436, -0·169 | < ·001 |  |  |
| *Sensitivity analysis 2 (without intercept): assessment of alcohol use (k = 16)* | | | | | |
| Aggregated consumption data | -0·083 | -0·207, 0·042 | ·192 | 99·9 | 4856·29 (< ·001) |
| Individual-level consumption data | -0·145 | -0·255, -0·035 | ·010 |  |  |
| *Sensitivity analysis 3 (without intercept): risk of bias (k = 16)* | | | | | |
| Low/moderate risk of bias | -0·083 | -0·207, 0·042 | ·042 | 99·9 | 4856·29 (< ·001) |
| Critical risk of bias | -0·145 | -0·255, -0·035 | ·010 |  |  |

The type of alcoholic beverage refers to the alcoholic beverage whose beverage-specific consumption served as the outcome variable in the original studies. 95% CI: confidence interval. RTD: ready-to-drink. Ref: reference. *Total variance. All studies relied on a longitudinal study design.

# Table S15. Results from the random-effects meta-analysis on the restrictions of alcohol sales on one day.

| **Model** | **Estimate** | **95% CI** | **p-value** | **Between-study heterogeneity** | | **Egger’s regression test for publication bias** |
| --- | --- | --- | --- | --- | --- | --- |
|  |  |  |  | **I^2^** | **Q (p-value)** | **z (p-value)** |
| Main model (k = 10) | -0·036 | -0·051, -0·022 | < ·001 | 75·1 | 36·19 (< ·001) | 0·60 (·547) |
| Leave-one-out sensitivity analysis | | | | | | |
| Carpenter et al_2009: alcohol | -0·039 | -0·05, -0·02 | < ·001 | 76·7 | 34·29 (< ·001) | · |
| Grönqvist et al_2014: alcohol | -0·036 | -0·05, -0·02 | < ·001 | 77·9 | 36·14 (< ·001) | · |
| Norström et al_2005: beer | -0·030 | -0·04, -0·02 | < ·001 | 33·0 | 11·95 (·154) | · |
| Norström et al_2005: spirits | -0·036 | -0·06, -0·02 | < ·001 | 77·9 | 36·19 (< ·001) | · |
| Norström et al_2005: wine | -0·038 | -0·06, -0·02 | < ·001 | 72·9 | 29·48 (< ·001) | · |
| Stehr et al_2007: beer | -0·037 | -0·05, -0·02 | < ·001 | 77·9 | 36·19 (< ·001) | · |
| Stehr et al_2007: spirits | -0·034 | -0·05, -0·02 | < ·001 | 76·1 | 33·41 (< ·001) | · |
| Yörük et al_2013: beer | -0·036 | -0·05, -0·02 | < ·001 | 77·9 | 36·16 (< ·001) | · |
| Yörük et al_2013: spirits | -0·040 | -0·05, -0·03 | < ·001 | 74·8 | 31·71 (< ·001) | · |
| Yörük et al_2013: wine | -0·037 | -0·05, -0·02 | < ·001 | 77·3 | 35·25 (< ·001) | · |

# Table S16. Sensitivity analysis: relative changes in alcohol consumption following the restriction of alcohol sales on one day.

| **Outcome variable: relative change in alcohol consumption** | | | | **Between-study heterogeneity** | |
| --- | --- | --- | --- | --- | --- |
| **Predictor** | **Estimate** | **95% CI** | **p-value** | **I^2^ (%)*** | **Q (p-value)** |
| *Sensitivity analysis 1 (meta-regression without intercept): beverage-specific or overall consumption (k = 10)* | | | | | |
| Alcohol consumption (not beverage-specific) | -0·025 | -0·061, 0·010 | ·163 | 76·2 | 14·14 (·028) |
| Beer-specific consumption | -0·056 | -0·081, -0·030 | < ·001 |  |  |
| Wine-specific consumption | -0·017 | -0·048, 0·014 | ·276 |  |  |
| Spirits-specific consumption | -0·032 | -0·056, -0·008 | ·010 |  |  |
| *Sensitivity analysis 2: meta-analysis, excluding one study with a critical risk of bias (k = 7)* | -0·030 | -0·047, 0·012 | ·001 | 31·8 | 8·80 (·185) |
| *Sensitivity analysis 3: meta-analysis excluding one study with a repeated cross-sectional study design (k = 9)* | -0·039 | -0·053, -0·024 | < ·001 | 76·7 | 34·29 (<·001) |
| *Sensitivity analysis 4: meta-analysis including only studies that tested the impact of permitting alcohol sales on one additional day a week*** | 0·034 | 0·019, 0·049 | < ·001 | 79·1 | 33·41 (<·001) |

NOS: Newcastle-Ottawa scale for cohort studies to access risk of bias. 95% CI: confidence interval. *Total variance. **only studies that tested the impact of permitting Sunday sales were included; thus, the pooled effect size describes the increase in consumption when Sunday sales were permitted. By restricting the meta-analysis to studies that tested the impact of permitting alcohol sales on one additional day a week (sensitivity analysis 4), the pooled *increase* in alcohol consumption was 3·4% (95% CI: 1·9%, 4·9%). This is similar to the observed *decrease* in consumption levels in the only study included that tested the impact of an alcohol sales ban (beer: 3·4%, spirits: -7·2%).^15^

# Figure S1. Funnel plot for the meta-regression model on the impact of alcohol tax changes on relative alcohol consumption changes.

# Figure S2. Funnel plot for the meta-analysis on introducing MUP, impact on alcohol consumption within one year.

# Figure S3. Funnel plot for meta-analysis on the restriction of alcohol sales on one day.

# References

1 Alexeev S, Weatherburn D. The Australian ready-to-drink beverages tax missed its target age group. *Int J Drug Policy* 2021; **95**: 103399.

2 O’Brien JW, Tscharke BJ, Bade R, *et al.* A wastewater‐based assessment of the impact of a minimum unit price (MUP) on population alcohol consumption in the Northern Territory, Australia. *Addiction* 2022; **117**: 243–9.

3 Vandenberg B, Jiang H, Livingston M. Effects of changes to the taxation of beer on alcohol consumption and government revenue in Australia. *Int J Drug Policy* 2019; **70**: 1–7.

4 Chikritzhs TN, Dietze PM, Allsop SJ, Daube MM, Hall WD, Kypri K. The “alcopops” tax: heading in the right direction. *Med J Aust* 2009; **190**: 294–5.

5 Doran CM, Digiusto E. Using taxes to curb drinking: A report card on the Australian government’s alcopops tax: Report on the Australian alcopops tax. *Drug Alcohol Rev* 2011; **30**: 677–80.

6 Rehm J, Tran A, Gobina I, *et al.* Do alcohol control policies have the predicted effects on consumption? An analysis of the Baltic countries and Poland 2000-2020. *Drug Alcohol Depend* 2022.

7 Chung VCH, Yip BHK, Griffiths SM, *et al.* The impact of cutting alcohol duties on drinking patterns in Hong Kong. *Alcohol Alcohol* 2013; **48**: 720–8.

8 Gehrsitz M, Saffer H, Grossman M. The effect of changes in alcohol tax differentials on alcohol consumption. *J Public Econ* 2021; **204**: 104520.

9 Saffer H, Gehrsitz M, Grossman M. The effects of alcohol excise tax increases by drinking level and by income level. Cambrige, MA: National Bureau of economic Research, 2022 http://www.nber.org/papers/w30097.

10 Tran A, Jiang H, Kim KV, *et al.* Predicting the Impact of Alcohol Taxation Increases on Mortality—A Comparison of Different Estimation Techniques. *Alcohol Alcohol* 2022; **57**: 500–7.

11 Subbaraman MS, Mulia N, Kerr WC, Patterson D, Karriker‐Jaffe KJ, Greenfield TK. Relationships between US state alcohol policies and alcohol outcomes: differences by gender and race/ethnicity. *Addiction* 2020; **115**: 1285–94.

12 Freeman DG. Beer in Good Times and Bad: A U.S. State-Level Analysis of Economic Conditions and Alcohol Consumption. *J Wine Econ* 2011; **6**: 231–51.

13 An R, Sturm. Does the response to alcohol taxes differ across racial/ethnic groups? Some evidence from 1984-2009 Behavioral Risk Factor Surveillance System. *J Ment Health Policy Econ* 2011; **14**: 13–23.

14 Nelson JP. How Similar are Youth and Adult Alcohol Behaviors? Panel Results for Excise Taxes and Outlet Density. *Atl Econ J* 2008; **36**: 89–104.

15 Stehr M. The Effect of Sunday Sales Bans and Excise Taxes on Drinking and Cross—Border Shopping for Alcoholic Beverages. *Natl Tax J* 2007; **60**: 85–105.

16 Dávalos ME, Fang H, French MT. Easing the pain of an economic downturn: macroeconomic conditions and excessive alcohol consumption. *Health Econ* 2012; **21**: 1318–35.

17 Khaltourina D, Korotayev A. Effects of Specific Alcohol Control Policy Measures on Alcohol-Related Mortality in Russia from 1998 to 2013. *Alcohol Alcohol* 2015; **50**: 588–601.

18 Heeb J-L, Gmel G, Zurbrügg C, Kuo M, Rehm J. Changes in alcohol consumption following a reduction in the price of spirits: a natural experiment in Switzerland: Reduction in the price of spirits. *Addiction* 2003; **98**: 1433–46.

19 Kuo M, Heeb J-L, Gmel G, Rehm J. Does Price Matter? The Effect of Decreased Price on Spirits Consumption in Switzerland. *Alcohol Clin Exp Res* 2003; **27**: 720–5.

20 Gmel G, Wicki M, Rehm J, Heeb J-L. Estimating regression to the mean and true effects of an intervention in a four-wave panel study. *Addiction* 2007; **103**: 32–41.

21 Sornpaisarn B. The Effectiveness of an Alternative Alcohol Taxation Method in a Middle-Income County: A Case Study of Thailand. 2013.

22 Stockwell T, Auld MC, Zhao J, Martin G. Does minimum pricing reduce alcohol consumption? The experience of a Canadian province: Does minimum pricing reduce alcohol consumption? *Addiction* 2012; **107**: 912–20.

23 Taylor N, Miller P, Coomber K, *et al.* The impact of a minimum unit price on wholesale alcohol supply trends in the Northern Territory, Australia. *Aust N Z J Public Health* 2021; **45**: 26–33.

24 Coomber K, Miller P, Taylor N, *et al.* Investigating the introduction of the alcohol minimum unit price in the Northern Territory (Final report). Geelong: Deakin University, 2020.

25 Stockwell T, Zhao J, Giesbrecht N, Macdonald S, Thomas G, Wettlaufer A. The Raising of Minimum Alcohol Prices in Saskatchewan, Canada: Impacts on Consumption and Implications for Public Health. *Am J Public Health* 2012; **102**: e103–10.

26 O’Donnell A, Anderson P, Jané-Llopis E, Manthey J, Kaner E, Rehm J. Immediate impact of minimum unit pricing on alcohol purchases in Scotland: controlled interrupted time series analysis for 2015-18. *BMJ* 2019; **366**: l5274.

27 Anderson P, O’Donnell A, Kaner E, Llopis EJ, Manthey J, Rehm J. Impact of minimum unit pricing on alcohol purchases in Scotland and Wales: controlled interrupted time series analyses. *Lancet Public Health* 2021; : S2468266721000529.

28 Rehm J, O’Donnell A, Kaner EFS, Jane LLopis E, Manthey J, Anderson P. Differential impact of minimum unit pricing on alcohol consumption between Scottish men and women: controlled interrupted time series analysis. *BMJ Open* 2022; **12**: e054161.

29 Llopis EJ, O’Donnell A, Anderson P. Impact of price promotion, price, and minimum unit price on household purchases of low and no alcohol beers and ciders: Descriptive analyses and interrupted time series analysis of purchase data from 70, 303 British households, 2015–2018 and first half of 2020. *Soc Sci Med* 2021; **270**: 113690.

30 Robinson M, Mackay D, Giles L, Lewsey J, Richardson E, Beeston C. Evaluating the impact of minimum unit pricing (MUP) on off‐trade alcohol sales in Scotland: an interrupted time–series study. *Addiction* 2021; **116**: 2697–707.

31 Xhurxhi IP. The early impact of Scotland’s minimum unit pricing policy on alcohol prices and sales. *Health Econ* 2020; **29**: 1637–56.

32 Stevely A, Mackay D, Hernández A, Meier P, Sasso A, Holmes J. WP3: The impact of MUP on harmful drinking in the general population: an interrupted time series analysis. In: Holmes J, ed. Evaluating the impact of Minimum Unit Pricing in Scotland on people who are drinking at harmful levels. Edinburgh, UK: Public Health Scotland, 2022. https://www.drugsandalcohol.ie/36398/1/PHS_Evaluating-the-impact-of-minimum-unit-pricing-in-scotland-report.pdf (accessed Oct 12, 2022).

33 Giles L, Robinson M, Beeston C. Minimum Unit Pricing (MUP) for alcohol evaluation. Sales-based consumption: a descriptive analysis of one-year post-MUP off-trade alcohol sales data. Edinburgh, UK: NHS Health Scotland, 2019 http://www.healthscotland.scot/media/2954/c-users-kims-desktop-sales-based-consumption-descriptive-analysis-of-one-year-post-mup-off-trade-alcohol-sales-data.pdf.

34 Kolosnitsyna M, Sitdikov M, Khorkina N. Availability restrictions and alcohol consumption: A case of restricted hours of alcohol sales in Russian regions. *Int J Alcohol Drug Res* 2014; **3**. DOI:10.7895/ijadr.v3i3.154.

35 Bassols NM, Castello JV. Bar opening hours, alcohol consumption and workplace accidents. *Labour Econ* 2018; **53**: 172–81.

36 Hough M, Hunter G. The 2003 Licensing Act’s impact on crime and disorder: An evaluation. *Criminol Crim Justice* 2008; **8**: 239–60.

37 Carpenter CS, Eisenberg D. Effects of Sunday Sales Restrictions on Overall and Day-Specific Alcohol Consumption: Evidence From Canada. *J Stud Alcohol Drugs* 2009; **70**: 126–33.

38 Norström T, Skog O-J. Saturday opening of alcohol retail shops in Sweden: an impact analysis. *J Stud Alcohol* 2003; **64**: 393–401.

39 Norström T, Skog O-J. Saturday opening of alcohol retail shops in Sweden: an experiment in two phases. *Addiction* 2005; **100**: 767–76.

40 Grönqvist H, Niknami S. Alcohol availability and crime: Lessons from liberalized weekend sales restrictions. *J Urban Econ* 2014; **81**: 77–84.

41 Yörük BK. Legalization of Sunday alcohol sales and alcohol consumption in the United States: Sunday alcohol sales. *Addiction* 2014; **109**: 55–61.
